# De novo design of potent inhibitors of clostridial family toxins

**DOI:** 10.1073/pnas.2509329122

**Published:** 2025-09-22

**Authors:** Robert J. Ragotte, Huazhu Liang, John Tam, Sean Miletic, Jacob M. Berman, Roger Palou, Connor Weidle, Zhijie Li, Matthias Glögl, Greg L. Beilhartz, Kenneth D. Carr, Andrew J. Borst, Brian Coventry, Xinru Wang, John L. Rubinstein, Mike Tyers, Daniel Schramek, Roman A. Melnyk, David Baker

**Affiliations:** ^a^Department of Biochemistry, University of Washington, Seattle, WA 98195; ^b^Institute for Protein Design, University of Washington, Seattle, WA 98195; ^c^Molecular Medicine Program, The Hospital for Sick Children, Toronto, ON, Canada M5G 0A4; ^d^Department of Biochemistry, University of Toronto, Toronto, ON M5S 1A8, Canada; ^e^Centre for Molecular and Systems Biology, Lunenfeld-Tanenbaum Research Institute, Mount Sinai Hospital, Toronto, ON M5G 1X5, Canada; ^f^HHMI, University of Washington, Seattle, WA 98195; ^g^Department of Medical Biophysics, University of Toronto, Toronto, ON, Canada M5G 1L7; ^h^Department of Molecular Genetics, University of Toronto, Toronto, ON, Canada M5S 1A8

**Keywords:** *C. difficile*, tcdb, tcsl, protein design, cryo-EM

## Abstract

*Clostridioides difficile* remains a leading cause of hospital-acquired infections, with its primary virulence factor, toxin B (TcdB), responsible for severe colitis and recurrent disease. The closely related toxin, TcsL, from *Paeniclostridium sordellii*, causes a rarer but often fatal toxic shock syndrome, particularly in gynecological and obstetric contexts. We report the de novo design of small protein minibinders that directly neutralize TcdB and TcsL by preventing their entry into host cells. Using deep learning and Rosetta-based approaches, we generated high-affinity minibinders that protect cells from intoxication with picomolar potency and, in the case of TcsL, prolonged survival following lethal toxin challenge in mice. The designed proteins against TcdB demonstrate exceptional stability in proteolytic and acidic environments, making them well-suited for oral delivery—a valuable feature for treating *C. difficile* infections localized to the gastrointestinal tract. For TcsL, potent inhibitors were identified from 48 initial designs and 48 optimized designs, highlighting the potential of computational design for rapidly developing countermeasures against life-threatening bacterial toxins.

## Significance

*Clostridioides difficile* infection (CDI) is a major public health concern with over half a million cases in the United States annually resulting in ~30,000 deaths. Current therapies are inadequate and frequently result in cycles of recurrent infection. Using de novo protein design, w developed small protein inhibitors targeting two independent receptor binding sites on the toxin that drives pathology during CDI. We extend this approach to develop inhibitors of TcsL, a related toxin that causes highly lethal toxic shock with no effective treatments, and show that these inhibitors prolong survival in a murine lethal toxin challenge model.

*Clostridioides difficile* toxin B (TcdB) drives the pathology of *C. difficile* infection (CDI), a common and sometimes fatal nosocomial disease of the colon with 500,000 cases per year in the United States (1). A related toxin family member, TcsL, produced by *Paeniclostridium sordellii*, causes a rarer but highly lethal disease via systemic toxic shock, which primarily affects postpartum women (2–5). The varied clinical manifestations of these related toxins can be attributed to distinct cell tropism through interactions with specific host receptors (6–9). Currently, vancomycin and fidaxomicin are the standard-of-care for acute CDI, but perturbations of the microbiota can contribute to disease progression (10), resulting in a high rate of relapse and patients entering cycles of recurrent CDI (rCDI). Bezlotoxumab, a therapeutic monoclonal antibody targeting TcdB, reduces the rate of rCDI by ~10%, providing a strong biological basis for targeting TcdB neutralization during CDI (11, 12). However, given the high cost, complex route of administration (intravenous) and unknown efficacy during acute CDI, bezlotoxumab is only recommended for those at high risk of rCDI. It remains unclear how bezlotoxumab compares to fidaxomicin, a comparably priced but orally administered antibiotic that similarly reduces the rate of rCDI (13, 14). There is a need for new anti-TcdB therapeutics that are orally available, act directly in the site of infection, are not cost-prohibitive and could be administered prophylactically during outbreaks without substantial risk of driving antibiotic resistance. For *P. sordellii* infection, the high mortality rate highlights a lack of effective therapeutic interventions for the associated toxic shock syndrome (3). Pathogenic *P. sordellii* strains lacking TcsL production show minimal disease symptoms in animal models (2), validating TcsL as a target for preventing and treating toxic shock syndrome.

We reasoned that de novo designed miniprotein inhibitors could help address unmet clinical needs for treating clostridial toxin-associated disease. Miniproteins have advantages over monoclonal antibodies in ease of production, thermostability, and protease stability while retaining high affinity and specificity for their targets (15–18). We set out to design small proteins that precisely target sites on clostridial toxins that engage cell surface receptors, thereby preventing the toxins from entering cells.

## Inhibition of the Frizzled/TFPI Binding Site.

**Fig. 1.**
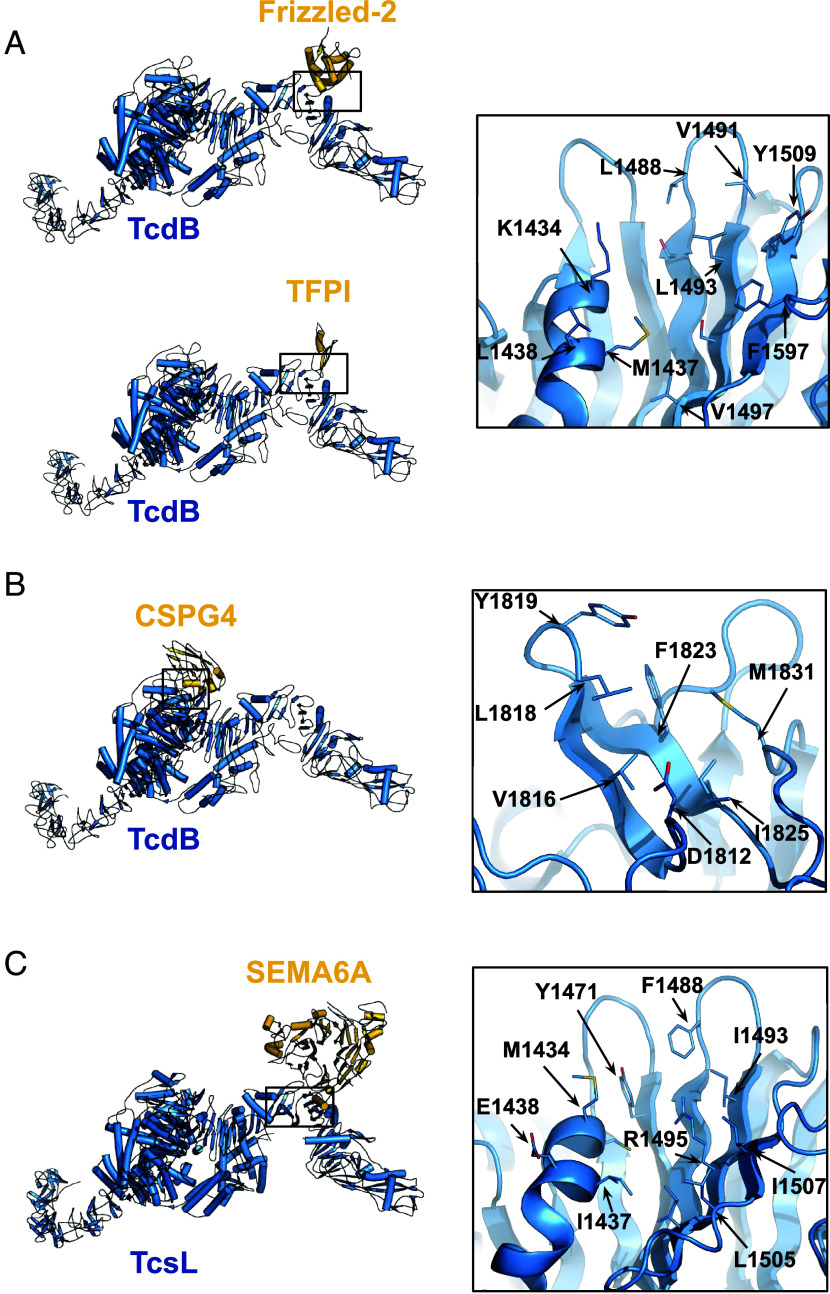
Composite models of clostridial toxin-host cell receptor complexes. Inset boxes highlight the residues targeted during design. (*A*) TcdB [PDB 6OQ5 (20)] with Frizzled-2 [PDB 6C0B (8)] and TFPI [PDB 7V1N (7)]. (*B*) TcdB [PDB 6OQ5 (20)] with CSPG4 [PDB 7ML7 (6)]. (*C*) TcsL [PDB 8JB5 (21)] with SEMA6A [PDB 6WTS (9)].

Receptor usage patterns of TcdB differ between variants of the toxin. TcdB uses a receptor binding site in the distal RBD that can interact with Frizzled-1/2/7 (8) or TFPI (7), as illustrated in Fig. 1*A*. A second receptor binding site is located in the toxin core where the toxin can bind to host CSPG4 (6), used alongside Frizzled-1/2/7 or independent of Frizzled in the case of TcdB2 (19) (Fig. 1*B*).

**Fig. 2.**
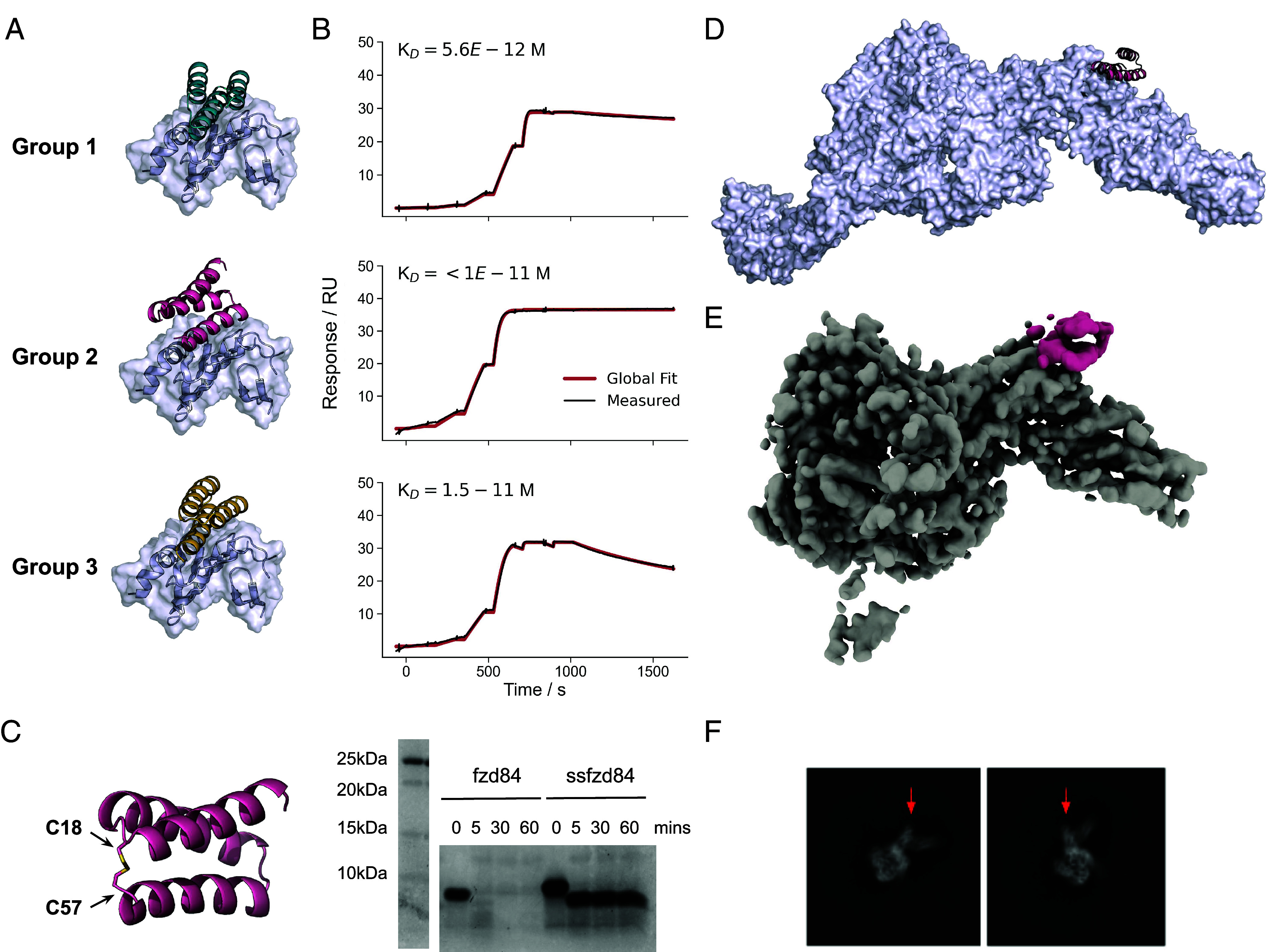
Design of anti-TcdB Frizzled-blocking minibinders. (*A*) Design models of three high affinity minibinder families. (*B*) Corresponding single cycle kinetic analysis of a design from each family (fzd13 from group 1, fzd48 from group 2 and fzd24 from group 3) with TcdB RBD captured on chip and a 6-step 4-fold dilution series starting at 100 nM. (*C*) Disulfide stabilization of fzd84 minibinder with the location of the disulfide introduced (*Left*) and a coomassie stained SDS-PAGE after a time course incubation in simulated intestinal fluid (with 0.1 mg/mL of trypsin and chymotrypsin) at 37 °C (*Right*). (*D*) Design model of fzd48 in complex with the full-length toxin from PDB 6OQ5. (*E*) Segmented cryoEM map of TcdB in complex with fzd48 (in pink). (*F*) Example class averages with arrows indicating additional density due to fzd48 binding.

We began by targeting the TcdB Frizzled/TFPI binding interface using a design pipeline integrating Rosetta- and deep learning-based methods (16, 22). We generated docks from 3-helical and 4-helical bundle libraries using RifGen and RifDock focused on hydrophobic residues at the binding interface (Fig. 1*A*). We then assigned sequences to the docks using ProteinMPNN and filtered the designs using AlphaFold2 initial guess (22, 23). 15,000 designs were screened for binding using yeast surface display followed by optimization through site saturation mutagenesis (16) (*SI Appendix*, Fig. S1). After sequence optimization, the highest affinity binders were expressed in *Escherichia coli,* and three sets of designs (each resulting from a single initial dock) had monodisperse SEC traces and high affinity for their target sites (Fig. 2*A* and *SI Appendix*, Fig. S2). These are defined as group 1, group 2, and group 3 designs. Designs within each group originate from the same initial dock (Fig. 2*A*). The sequences of the parent and optimized designs are provided in *SI Appendix*, Table S1.

For group 2 designs, accurate k_off_ could not be determined even with dissociation times > 1 h, as minimal dissociation was detected (Fig. 2*B* and *SI Appendix*, Fig. S3), suggesting K_D_s in the low pM range (*SI Appendix*, Table S2). In neutralization assays using CSPG4-deficient Vero cells, fzd5 (group 1), fzd48 (group 2), and fzd84 (group 2) had IC50s of 31 (95% CI 22 to 48), 133 (95% CI 97 to 187) and 61 (95% CI 38 to 101) pM in the presence of 1.5 pM toxin, respectively (Fig. 4 *A* and *B*).

The structure of fzd48 in complex with TcdB was determined by electron cryomicroscopy (cryoEM). The binding epitope and orientation match the design model (Fig. 2 *D*−*F* and *SI Appendix*, Fig. S4 and Table S3). At 4.6Å resolution, we could clearly resolve the secondary structure of fzd48 with density for all three helices at its intended target site on the knuckle of the RBD (Fig. 2 *D*−*F*). In the design model of fzd48, W47 extends into the hydrophobic pocket formed from the coming together of the kinked α-helix and the 5-stranded β-sheet in the RBD that composes the bulk of the interface. K35 and K54 flank this hydrophobic core of the interface, potentially forming salt bridges with neighboring D1490 and E1547 on TcdB (the limited resolution of the cryoEM map prevented building and assessment of sidechain accuracy).

TcdB acts in the colonic lumen and hence is only amenable for treatment by protein-based biologics if an inhibitor can survive in the gastrointestinal tract without extensive proteolysis. To improve the protease stability of the designs (which were degraded rapidly in simulated intestinal fluid (Fig. 2*C* and *SI Appendix*, Fig. S5), we introduced disulfides into the fzd group 1 and group 2 designs. In group 2, the disulfide connects a loop between the first and second helix with the C terminus, effectively locking the structure together (Fig. 2*C*). Both group 1 and group 2 disulfide stabilized designs retained their binding activity and were highly resistant to degradation in simulated intestinal fluid for 1 h at 37 °C, the longest time tested (Fig. 2*C* and *SI Appendix*, Fig. S5). ssfzd84, a group 2 design with a C18-57 disulfide, showed no discernible degradation (except for the loss of the C-terminal SNAC and his tags) and had an IC50 of 24 (95% CI 14 to 40) pM in the TcdB neutralization assay (Fig. 4*C*).

## Inhibition of the CSPG4 Binding Site.

**Fig. 3.**
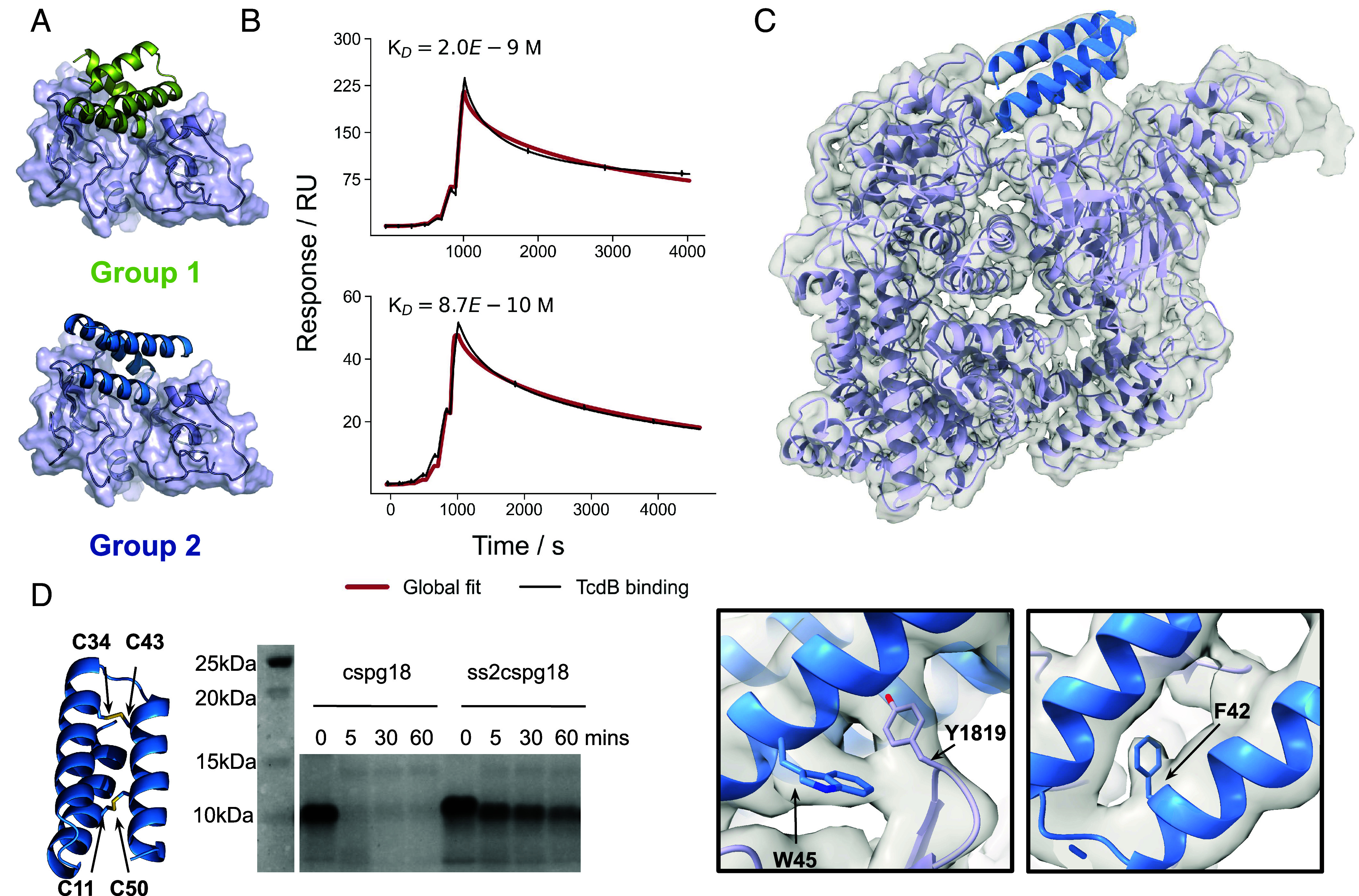
Design of anti-TcdB CSPG4-blocking minibinders. (*A*) Design models of the two CSPG4-blocking minibinder families. (*B*) Single cycle kinetic analysis of a group 1 (design cspg4) and group 2 (design ss2cspg18) minibinder amine conjugated to the surface across a 4-fold, 6-step dilution series of full-length TcdB with an upper concentration of 100 nM. (*C*) The design model docked into the CryoEM density map showing agreement between the observed density and the design. *Lower Inset* boxes highlight specific side chains on the design model resolved at the target:binder interface. (*D*) Disulfide stabilization of CSPG4-blocking minibinders with the location of the two disulfides introduced to make ss2cspg18 (*Left*) and a coomassie stained SDS-PAGE after a time course incubation in simulated intestinal fluid (with 0.1 mg/mL of trypsin and chymotrypsin) at 37 °C with cspg18 and the dual disulfide version (ss2cspg18) (*Right*).

**Fig. 4.**
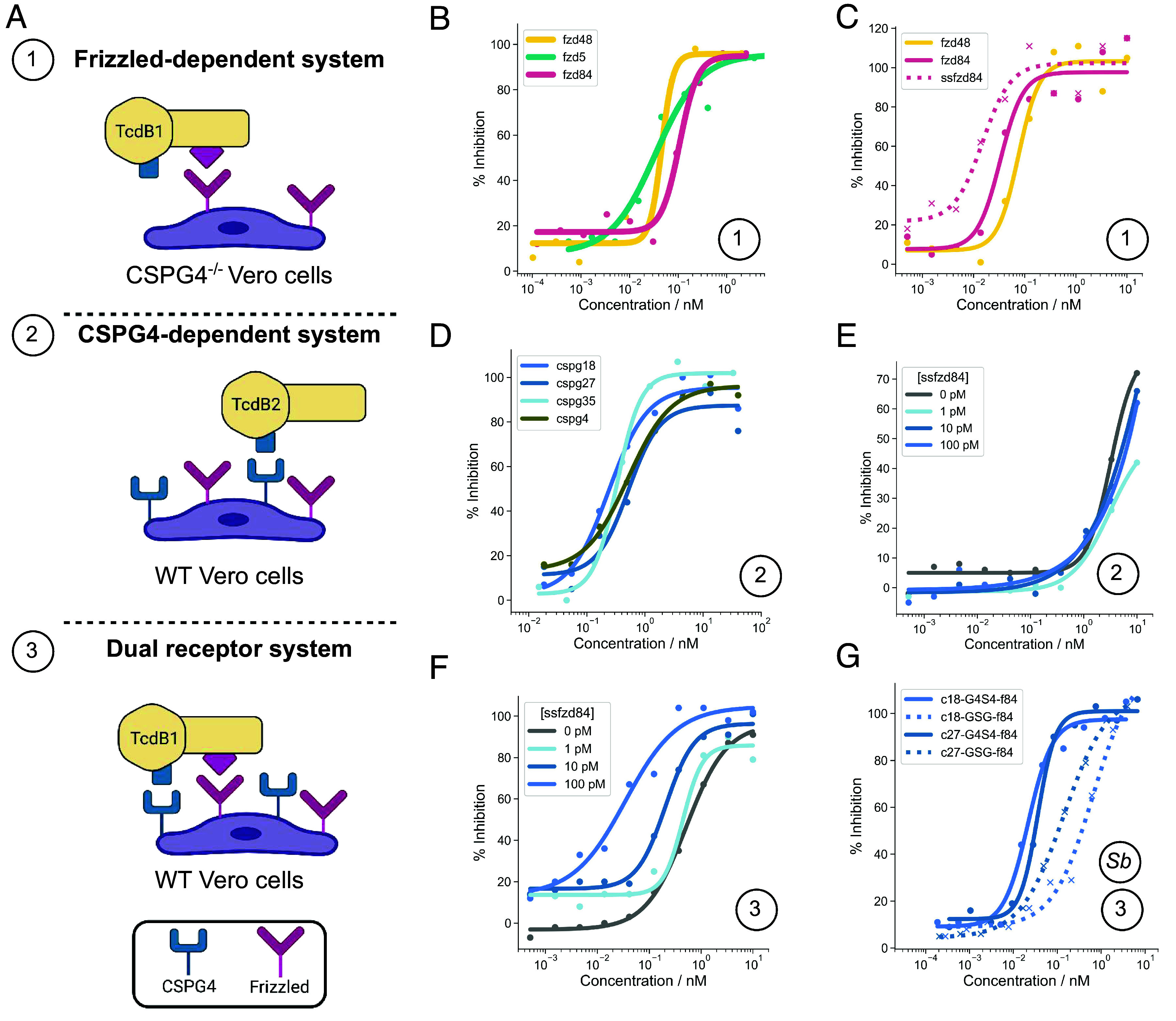
Minibinder neutralization of TcdB in Vero cells. (*A*) Overview of the three experimental set-ups used to generate the neutralization data for the different classes of binders alone and in combination. (*B*) Sequence optimized fzd binders from group 1 (fzd5) and group 2 (fzd48, fzd84). (*C*) Protease resistant, sequence optimized fzd binder ssfzd84 compared with the protease susceptible designs. (*D*) Sequence optimized cspg designs from group 1 (cspg4) and group 2 (cspg18, cspg27, cspg35). (*E*) Titration of disulfide stabilized ss2cspg18 (*x*-axis) in the presence of four concentrations of ssfzd84 in the CSPG4-dependent system (*F*) Titration of ss2cspg18 (*x*-axis) with four concentrations of ssfzd84 in the dual receptor system. (*G*) Neutralization activity of fzd-cspg binder fusions secreted by *S. boulardii*. Fusion constructs between ss2cspg18 (c18) and ss2cspg27 (c27) fused to ssfzd84 (f84) with either a short GSG linker that should not allow simultaneous receptor engagement (dashed) or a longer (G4S)_4_ linker that should enable simultaneous binding at the two sites (solid). In all cases, a single example from independent replicates is shown. Reported IC50 values (in text) are the average across independent replicates. All response curves plotted on the same axes were run in the same experiment. The circled number corresponds to the experiment system in panel *A*, while *Sb* indicates the use of *S. boulardii* secreted protein.

We applied the same approach to the CSPG4 binding site which was more challenging due to the complex topology of the binding site (Fig. 1*B*). Using the same Rifgen/Rifdock approach followed by ProteinMPNN, sequence optimization (*SI Appendix*, Fig. S6), and the addition of a single disulfide, we achieved low or subnanomolar binding against the CSPG4 binding site from two different families of designs, designated group 1 and 2 (Fig. 3 *A* and *B*, S8C with sequence information in *SI Appendix*, Table S1 and kinetic parameters in *SI Appendix*, Table S2). Using TcdB2, which preferentially uses CSPG4 for cell entry (Fig. 4*A*), we found that these molecules can protect against cytotoxicity with IC50s of 238 (95% CI 43 to 495), 520 (95% CI 85 to 1,320), and 332 (95% CI 240 to 444) pM for group 2 designs cspg18, cspg27, and cspg35, respectively, after 48 h in the presence of 0.1 pM TcdB (Fig. 4*D*). Although these designs already included one disulfide bond, they remained susceptible to proteolytic degradation. We stabilized these designs by introducing a second disulfide bond (Fig. 3*D*). After 1 h in the presence of simulated intestinal fluid, intact protein remained compared to complete digestion by 5 min for the single disulfide parent of the binder (Fig. 3*D* and *SI Appendix*, Fig. S8) with no loss in neutralization potency in vitro (*SI Appendix*, Fig. S8).

We determined the structure of a cspg group 2 binder, cspg67, in complex with TcdB by cryo-EM (Fig. 3*C* and *SI Appendix*, Table S3). Helices two and three engage with a hydrophobic patch formed from a small two stranded sheet adjacent the native CSPG4 binding cleft (V1816, L1818, Y1819, F1823) and M1831 from a neighboring loop, binding to TcdB in the open conformation. The loop connecting helices 2 and 3 extends into the CSPG4 binding cleft where the C terminus of the second helix engages with the polar target surface, primarily through K32 and R36 that flank D1812 on TcdB. Because the binding site is in the central GTD and CPD domains, as opposed to the distal DRBD, we were able to resolve bulky side chains (F42, W45 on the binding molecule), which closely align with the design model side chain conformations (Fig. 3*C*).

To explore the effects of using fzd and cspg minibinders in combination, we tested a titration series of ss2cspg18 (the disulfide stabilized version of cspg18) with 4 concentrations of ssfzd84 (0, 1 pM, 10 pM, 100 pM) in both a CSPG4-dependent set-up using TcdB2 at its EC99 (0.1 pM), and a dual CSPG4- Frizzled-dependent set-up using TcdB1, also at its EC99 (0.1 pM), on Vero cells expressing both receptors (Fig. 4*A*). In the dual receptor dependent system, the combination of the Frizzled- and CSPG4-blocking minibinders together resulted in an enhanced dose-dependent inhibition of cell toxicity with increasing concentration of ssfzd84, as the curves shift to the left from an IC50 of 520 (95% CI 416 to 671) pM to 35 pM (95% CI 3.7 to 86) pM at 100 pM of ssfzd84 (Fig. 4*F*). There is also an increase of maximum protection to 100%, which is not achieved by ss2cspg18 alone (Fig. 4*F*). These results indicate that combinations of both ss2cspg18 and ssfzd84 provide cooperative protection against TcdB cytotoxicity when both receptors can be used. In the CSPG4-dependent paradigm, there was no additional benefit of increasing ssfzd84 concentrations (Fig. 4*E*).

## Secretion of fzd/cspg Fusion Constructs in the Probiotic Yeast *S. boulardii*.

Producing biologics in situ at the desired site of action is appealing as it would enable continuous production of the biotherapeutic, rather than necessitating repeated oral dosing. *Saccharomyces boulardii* is a probiotic yeast strain that can transiently colonize the lower GI tract (24) that is not susceptible to antibiotic treatment, unlike other experimental bacterial delivery platforms, and has previously been used successfully to deliver antibody-like constructs to the GI tract (25). We therefore tested the production and secretion of minibinders in *S. boulardii*. In vitro, the disulfide bond stabilized monomeric minibinders against TcdB were secreted at concentrations up to 50 nM (*SI Appendix*, Fig. S9). A fusion of the lead CSPG4- and Frizzled-blocking minibinders was also efficiently secreted from *S. boulardii* (*SI Appendix*, Fig. S9). Supernatants from *S. boulardii* culture expressing this construct (ss2cspg18 and ssfzd84 fused with a (G4S)_4_ linker) neutralized the toxin with an IC50 of 22 pM against TcdB1, which can use either CSPG4 or Frizzled to enter cells (Fig. 4*G*). The same fusion linked with only GSG, which is too short to allow simultaneous engagement with both receptor interfaces on TcdB, had a substantially higher IC50 of 672 pM. Thus, avidity achieved by linking binding domains against different regions of the monomeric toxin can enhance activity. These results demonstrate that multiple minibinder formats can be efficiently produced and secreted by *S. boulardii,* setting the stage for miniprotein-based synthetic biotics as therapeutics in a variety of GI disease contexts.

## Neutralization of TcsL and Protection from Lethal Toxic Shock.

Given the success of neutralizing TcdB, we sought to design inhibitors of *P. sordellii* lethal toxin, TcsL. TcsL causes a highly lethal disease in humans and livestock. In humans, this primarily affects postpartum and postabortive women, the latter associated with the use of misoprostol/mifepristone (26).

**Fig. 5.**
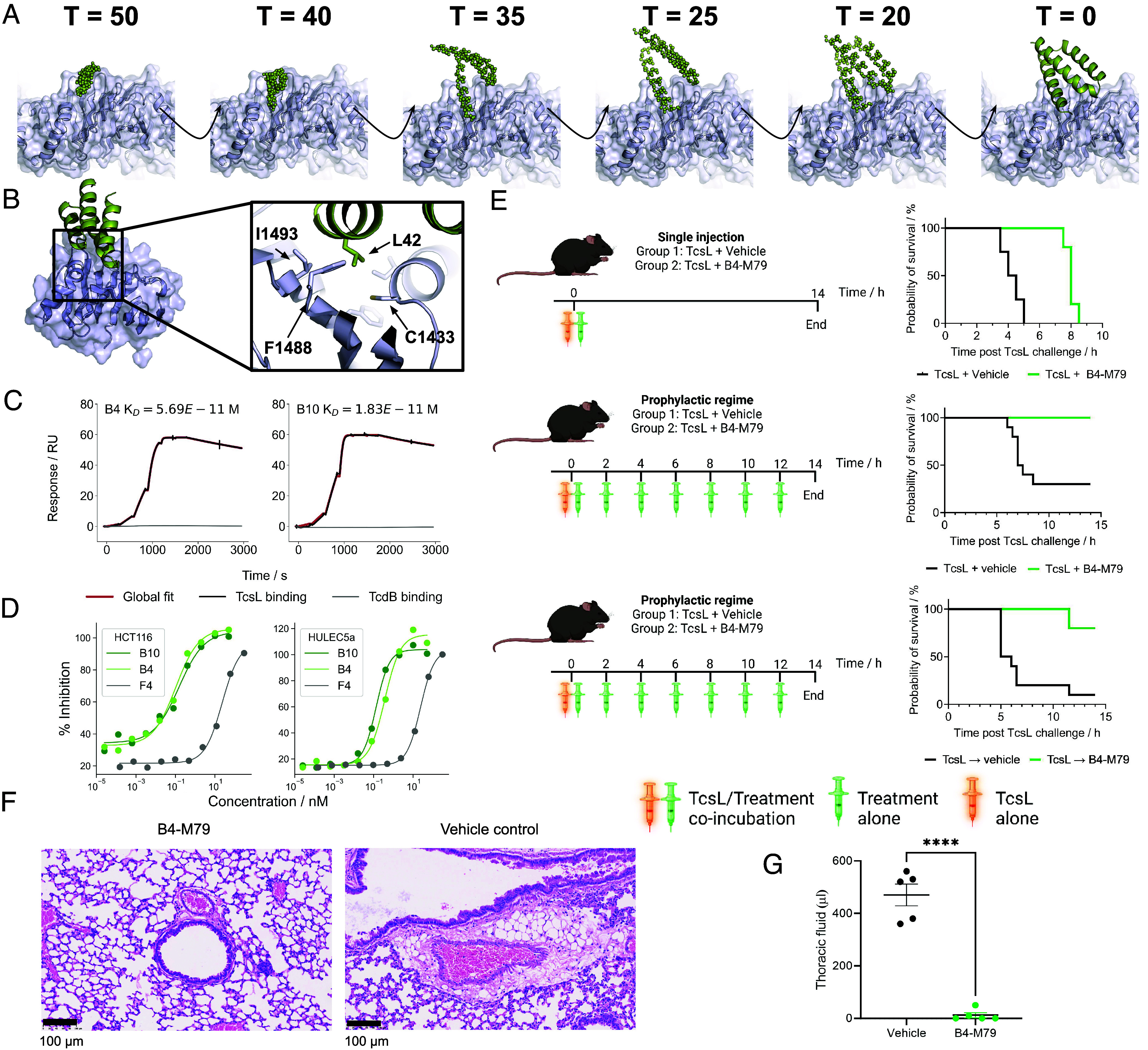
Design and characterization of TcsL-blocking minibinders. (*A*) RFdiffusion denoising trajectory starting from random noise placed above the target site at T = 50 to the fully denoised design at T = 0. (*B*) Design model of F4 binding to the RBD of TcsL. The inset box shows the hydrophobic pocket that accounts for the bulk of the interface. (*C*) Single cycle kinetic analysis with SPR of two of the optimized designs to TcsL (black) and TcdB (gray) with toxin RBD captured and a 6-step fourfold dilution series of the minibinder starting at 100 nM. (*D*) Neutralization of TcsL by parental (F4) and optimized (B4 and B10) designs on HCT116 cells (*Left*) and HULEC5a cells (*Right*). A single example from independent replicates is shown. Reported IC50 values (in text) are the average across independent replicates. All response curves plotted on the same axes were run in the same experiment. (*E*) Administration of a single bolus of TcsL alone or coincubated with a 1,000x molar excess of B4-M79. B4-M79 extended survival time from a median of 4.25 h to 8 h *P* = 0.0027 (log-rank test) (*Upper*). Administration of TcsL alone or coincubated with a 1,000x molar excess of B4-M79, followed by dosing with the same 1,000x molar excess (0.06 mg/kg) every 2 h, *P* = 0.0002 (*Middle*). Administration of TcsL followed by vehicle or B4-M79 1 h later, and repeated dosing every 2 h, *P* = 0.0012 (*Lower*). (*F*) Example histological images of the mouse lung from treatment group B with H&E stain showing the presence of edema in the vehicle control mice, characteristic of TcsL toxic shock. (*G*) Quantification of fluid in the lung of control mice vs treated mice in group B. Lines indicate mean ± SE. *P* < 0.0001 from a two-tailed unpaired *t* test.

Unlike TcdB, TcsL uses a single known receptor binding site to engage with SEMA6A and SEMA6B on the host cell surface, structurally similar to the TcdB-Frizzled interaction (Fig. 1*C*) (9, 27). We used the generative AI method RFdiffusion to design 55−65 residue miniprotein binders to the SEMA6A/B interface of TcsL beginning from randomly generated residue coordinates and then iteratively denoising over 50 time steps (a representative denoising trajectory is shown in Fig. 5*A*) (28). 10,000 diffusion trajectories were targeted to hydrophobic hotspot residues at the SEMA6A interface (Fig. 5*B*). Sequences that could fold into the intended structure and were compatible with target site binding were designed using ProteinMPNN (23) and the resulting designs filtered using AlphaFold2 initial guess (22). Overall, the design pipeline was similar to that used to generate the TcdB binding proteins, with the Rosetta-based scaffold docking replaced by RFdiffusion.

Because of the excellent in silico metrics, only 48 designs were produced in *E. coli* and screened for TcsL binding (*SI Appendix*, Fig. S10). The highest affinity, F4, bound with a K_D_ of 4 nM (*SI Appendix*, Table S2). F4 was further optimized using ProteinMPNN (*Materials and Methods*) (22) and 48 additional designs were experimentally tested (*SI Appendix*, Fig. S11). Two – B4 and B10 – were found to have very high affinities of 57 and 18 pM (Fig. 5*C* and *SI Appendix*, Table S2), though 11 others had subnanomolar binding affinity to the RBD (*SI Appendix*, Fig. S11*C*). For both B4 and B10, as well as the F4 parent, the bulk of the interface is centered around a hydrophobic pocket on the target site composed of C1433, I1434, I1437, and F1488 into which L42 on the binder extends (Fig. 5*B*). The ProteinMPNN optimized designs contain P > K and S > K substitutions that likely form salt bridges with neighboring glutamate residues on TcsL. Consistent with this binding epitope, B4 blocked SEMA6A binding to TcsL RBD in a competition assay (*SI Appendix*, Fig. S11*D*).

In a viability assay using HCT116 cells and 50 pM of TcsL (the EC99 of the toxin on this cell line), the IC50s for B4 and B10 were 238 (95% CI 122 to 476) and 210 (95% CI 51.6 to 8171) pM, respectively (Fig. 5*D*). Given that the primary target of TcsL during intoxication is lung vascular endothelial cells (29), we tested neutralization using the HULEC5a cell line which has elevated SEMA6A expression. Using 0.5 pM TcsL (the toxin EC90 on this cell line), B4 and B10 remained highly protective with IC50s of 248 (95% CI 88 to 706) and 157 (95% CI 85 to 292) pM (Fig. 5*D*). The designs bound specifically to TcsL, with no detectable binding to TcdB, despite 76% sequence identity between the two toxins (Fig. 5*C*).

Due to their small size, minibinders have a short half-life in circulation as they are rapidly removed by glomerular filtration. To overcome this, we evaluated the in vivo efficacy of B4 fused to the albumin binding domain M79 (30). This fusion did not affect binding affinity or neutralization (*SI Appendix*, Fig. S12). When TcsL was coadministered with a 0.06 mg/kg dose (1,000-fold molar excess compared to toxin) of B4-M79, we observed significant survival extension from a median of 4 h with the vehicle control to 8 h with the B4-M79 fusion (Fig. 5*E*). We next explored whether repeated dosing at 2 h intervals could further improve survival in both a prophylactic (coincubation of toxin and drug prior to administration) and therapeutic (toxin administration followed by drug administration 1 h later). In the prophylactic setting with repeated dosing at 0.06 mg/kg, mice that received the B4-M79 therapy were completely protected from death, with little to no indication of lung edema, as assessed by the collection of fluid from the lung and histology (Fig. 5 *F* and *G*). In the more stringent therapeutic setting 8/10 mice survived until the study end (14 h post toxin administration) with the other two animals exhibiting disease symptoms and becoming moribund at 11.5 h (Fig. 5*E*). In contrast, the median survival time of those receiving vehicle alone after toxin administration was 5.5 h (Fig. 5*E*). B4-M79 also could be lyophilized with no loss in neutralization, providing a viable pathway for long term storage (*SI Appendix*, Fig. S12).

## Discussion

Our designed binders provide a promising route for combatting the major health challenge presented by clostridial toxins. Therapeutics that neutralize toxins rather than directly killing pathogens should elicit diminished evolutionary pressure toward antimicrobial resistance, as the toxin is typically not necessary for survival (31). Targeting *C. difficile*-specific virulence factors is particularly appealing because antibiotic-induced disturbances to the microbiota contribute to disease progression and relapse (32). Effective CDI treatment through TcdB neutralization has the potential to improve both clinical outcomes for patients as well as antibiotic stewardship.

The possibility of a virulence-factor centered approach has been highlighted by the efficacy of bezlotoxumab, a TcdB targeting monoclonal antibody that reduces the rate of recurrent CDI (11). Despite the clinical benefit of bezlotoxumab treatment, uptake has been limited, largely due to cost (33) and the product was recently discontinued (34). Our designed miniproteins target two distinct sites and have very high affinity, protease resistance, and can be secreted from an *S. boulardii* probiotic drug delivery platform to neutralize TcdB. When fused together, these miniproteins show potent in vitro neutralization of the toxin in a dual-receptor dependent assay, with IC50s in the picomolar range. The ease of production, either as a recombinant protein or secreted from *S. boulardii*, potential for oral delivery and more targeted approach to CDI control (as opposed to conventional antibiotics) could enable these molecules to be administered prophylactically during *C. difficile* outbreaks to prevent symptom onset or to treat acute CDI at low cost. The viability of this approach will depend on the ability of these molecules to retain function within the gastrointestinal tract where these proteins will be subject to protease exposure. The stability of these molecules in simulated intestinal fluid containing high concentrations of trypsin and chymotrypsin is promising regarding their ability to neutralize TcdB in vivo; future work will evaluate this directly as both recombinant protein therapies and secreted from *S. boulardii* alongside administration of existing front-line antibiotics for CDI (vancomycin and fidaxomicin). Thus far, miniproteins have not been found to elicit strong immune responses in humans (35), but as with any new therapeutic it will be important to test the immunogenicity of these compounds and its relevance in the context of acute infection.

Alternative approaches to direct toxin neutralization such as nanobodies (36) and DARPins (37) and a bispecific antibody-like molecule (38) lack the control offered by computational design. The target sites of designed binders can be prespecified, avoiding dominance of clones that do not target the precise epitope of interest, which is a common challenge during selection. The TcsL case highlights that protein design methodology has advanced to enable isolation of high affinity binders from small screens (48 initial designs in this case) enabling the measurement of important biophysical properties of each design (soluble expression, aggregation state, binding kinetics) in a matter of days, compared to weeks to months to get equivalent data starting from a display library. Minibinders also have the advantage of interacting with the target through defined secondary structure, as opposed to loops, with the former being more readily imbued with protease stability. In recent years, increasingly complex surface topologies have been successfully targeted with designed minibinders (39), potentially overcoming a key drawback of DARPins, which favor particular structural motifs (40).

The advantages of de novo design are highlighted in a recent study by Lv et al., which combined a Rosetta-designed minibinder targeting the Frizzled site of TcdB with an engineered CSPG4 domain (38). Their binder showed cross-reactivity across TcdB variants 1 to 4 and in vivo efficacy in a colon-loop assay. We advance this approach by designing protease stable, high-affinity binders to both the Frizzled and CSPG4 interfaces, as well as to TcsL, a distinct and highly lethal clostridial toxin. By using a de novo designed binder rather than the CSPG4 domain, we avoidthe risk of off-target effects through the activation of host signaling pathways and/or the induction of anti-drug antibodies to endogenous CSPG4.

The design of high affinity inhibitors of TcsL from a total of 96 designs highlights how deep learning-based protein design can rapidly generate high affinity binders with in vivo efficacy. For *P. sordellii*, current treatments focus on clearing the infection with surgical removal of the affected tissue and the administration of broad-spectrum antibiotics to eliminate the pathogen (41, 42). The high (~70%) lethality highlights a lack of effective therapeutic intervention for this rare disease (5, 26, 42, 43). The in vivo efficacy of these molecules, and stability after lyophilization suggests they could have considerable therapeutic potential to treat this lethal disease. While the efficacy of the TcsL minibinders in preventing TcsL toxicity in vivo validates the minibinder-based toxin neutralization approach for clostridial-family toxins, for CDI it will be important to ensure that the minibinders retain activity at the site of action, and for the *S. boulardii* approach, that they are secreted in sufficient quantities in vivo. Moving forward, our minibinder design approach should be readily applicable to other infectious disease-associated toxins, and to other infectious disease targets, such as pathogen biofilms and inflammatory cytokines.

## Materials and Methods

### Design and Optimization of Frizzled- and CSPG4-Blocking Minibinders.

Docks of both Frizzled- and CSPG4-blocking minibinders were generated using a Rifgen-Patchdock-Rifdock procedure as described in depth previously (16, 44, 45). The input structure for Frizzled-blockers was 6C0B (8) with the Frizzled chain removed and the model cropped to remove the distal arms that were far from the Frizzled binding site (for computational efficiency). The docking procedure used a library of 30,000 helical bundles that were docked against hotspot residues M1437, L1483, L1488, V1491, Y1509, and F1597. For CSPG4-blocking binders, the input PDB used was 7ML7 (6), again with the receptor chain removed. The hotspot residues selected for the docking procedure were V1816, L1818, Y1819, F1823, and M1831. The Rifdock outputs then underwent sequence design using ProteinMPNN as described above with one sequence generated prior to FastRelax and one post FastRelax at temperature 0.001. For Frizzled blocking binders, a predicted local distance difference test (pLDDT) cutoff of 90 was used alongside a pairwise aligned error (PAE) interaction of < 8 to reach 15,000 designs. For CSPG4, only 2811 designs were ordered, all of which had a PAE interaction < 10.

Initial hits were validated for binding via BLI (fzd designs only) and TcdB neutralization assays (both fzd and cspg designs) and then underwent a site saturation mutagenesis screen via yeast surface display (described below). The most promising mutations, based on affinity improvement without increasing binding surface hydrophobicity, were selected to be included in a combination library which was ordered as an IDT ultramer with approximately 1E6 diversity. The combo libraries were assembled from two ultramers via PCR extension where necessary due to length constrictions and then cloned into yeast as described below. The sequences for the combo libraries are included in *SI Appendix*, Table S4. Group 2 CSPG4-blocking designs had a free cysteine inherited from the parental design. This was mutated to methionine or isoleucine (based on the SSM) prior to introducing the first disulfide bond (see below for disulfide stabilization).

### Design of SEMA6A-Blocking Minibinders.

Initial backbones were generated using RFdiffusion. The target structure was from the RBD of TcsL bound to SEMA6A [6WTS (9))] (but with the receptor chain removed) and hotspot residues F1488, I1493, L1505, and I1507 with RFdiffusion. 10,000 outputs were generated and then sequence designed using ProteinMPNN and FastRelax (22, 23), as above. One sequence from ProteinMPNN pre-FastRelax and one sequence post-FastRelax were generated for each design both at a temperature of 0.001. These designs were then filtered using a pLDDT > 90 cutoff and the top 48 designs with the lowest PAE interaction were ordered, corresponding to a cutoff of 5.7 (22).

During the second round of design, 10,000 new sequences of backbone of design F4 were generated at temperature 0.4 (no FastRelax was used here) and again filtered with 48 new designs ordered with a pLDDT cutoff of 96 and a PAE interaction cutoff of 4.5.

### Disulfide Stabilization of fzd and cspg Designs.

Disulfides were introduced using a previously published disulfide stapler (46) that can identify sites which accommodate disulfide bonds through searching 30,000 examples of disulfide bonds in native proteins. A single site was identified in each of the backbones for group 1 and group 2 fzd binders between residues 28 and 44 in group 1 and 18 and 57 in group 2. Within the group 2 cspg designs, possible disulfide sites were between residues 11 and 50, 23 and 53, and 34 and 43. Initial cspg designs included a single disulfide, but this did not yield protease resistance so two pairs of disulfide bonds were tested in each of the neutralizing designs. In each case, the disulfide stabilized designs were expressed at the 4 mL scale, as described below in the *E. coli* expression section. ssfzd designs expressed well in BL21 (NEB) whereas ss2cspg designs were expressed in T7 shuffle express (NEB).

### Electron Microscopy of TcdB Complexes.

#### CryoEM of TcdB:fzd48.

TcdB was thawed and complexed with 3 fold molar excess minibinder fzd48 at room temperature for 10 min before dilution to 0.81 mg/mL TcdB concentration in buffer [Tris/HCl pH 7.5 (25 mM) NaCl (150 mM)]. Samples applied onto freshly glow-discharged grids (−15 mA, 25 s, 0.39 mBar) 2/2 C-flat holey carbon grids in the chamber of a Mark IV Vitrobot, with humidity held at 100% and temperature at 22 °C. The wait time before blotting was 7.5 s, with a blot time of 7.0 s and a blot force of 0, after which the grid was plunge frozen in liquid ethane.

Cryo-EM grids were screened and data were collected on a ThermoFisher Titan Krios transmission electron microscope (FEI Thermo Scientific, Hillsboro, OR) operated at 300 kV and equipped with a Selectis energy filter and Gatan K3 direct detector. Data collection was automated with SerialEM (47). A total of 6713 movies were collected: 2,186 with no stage tilt, 3,873 at a 15^o^ stage tilt, and 654 at a 30° stage tilt. Movies were acquired in superresolution mode at a nominal magnification of 105,000x (0.4215 Å/pixel superresolution pixel), fractionated in 100 frames at 9.4 e^−^/Å^2^ /sec for a total exposure of ~47 e^−^/Å^2^ over 5.0 seconds. All data processing was carried out in cryoSPARC v4.4. Alignment of movie frames was performed using Patch Motion Correction (48). Defocus and astigmatism values were estimated using Patch CTF with default parameters.

Curate Exposures was used to remove 750 micrographs, leaving 5963 good micrographs. 1,068,213 particle images were initially selected using Blob Picker with a minimum and maximum particle diameter of 200 and 400 Å extracted in 920 × 920 pixel boxes and Fourier cropped to 460 × 460 pixels. Following 2D classification the five best classes, containing 39,344 particle images, were used to train Topaz on all 5963 good micrographs. Topaz extract was used to select 2,755,304 particle images, extracted in 920 × 920 pixel boxes and Fourier cropped to 460 × 460 pixels. This procedure was followed by 2D classification with 300 classes, 86 iterations 11 of which were final iterations using all particles. The 15 best classes, containing a total of 148,808 particle images, were subjected to 2D classification again using 50 classes. The best 23 classes, containing a total of 108,076 particle images, were used for 3D ab initio structure determination with C1 symmetry. The initial structure was refined with Non-Uniform Refinement (49) with C1 symmetry for a final global resolution estimate of 4.6 Å, including correction for the effects of masking. The final map was sharpened using DeepEMhancer (50). 3D maps for the half maps, final unsharpened maps, and the final sharpened maps were deposited in the EMDB under accession number EMD-45739. Local resolution estimation was run on the final Non-Uniform Refinement map.

The published crystal structure of TcdB (pdb ID: 6OQ5) (20) was used as an initial reference for building the final cryoEM structure. The model was fit into density using UCSF ChimeraX (51). Isolde (52) in UCSF ChimeraX and Coot (53) was used to better fit portions of the model to the map. Phenix (54) was used to trim the model to polyA, before further refinement in Coot and Isolde. Phenix and MolProbity (55) were used to assess model quality throughout. The de novo minibinder fzd48 was added last to remove bias, with further refinement in Coot and Isolde. Figures were generated using UCSF ChimeraX. The final structure was deposited in the PDB under number 9CM5.

#### CryoEM of TcdB:cspg67.

For the CryoEM of TcdB and CSPG4-blockers, cspg67 belonging to the group 2 family of cspg designs was used. cspg67 is a sequence variant of the group 2 backbone. Purified TcdB in a buffer containing 50 mM NaCl and 20 mM tris at pH 8 was mixed with cspg67 at a 1:1 molar ratio and incubated on ice until use. Three microliters of the mixture was applied to nanofabricated holey gold grids (56) which were previously glow discharged in 40 mbar air for 40 s with 25 mA current. The sample was applied in the environmental chamber of a Leica EM GP2 plunge freezing device (Leica Microsystems) at 4 °C and ~80% humidity, with a 30 s preincubation time and a 2 to 3 s blot time before plunge freezing in liquid ethane.

The specimen was imaged with a Titan Krios G3 300 kV cryoelectron microscope equipped with a Falcon 4i direct detector (ThermoFisher Scientific). The specimen was imaged at a nominal magnification of 75,000x, corresponding to a calibrated pixel size of 1.03 Å. Data collection was automated with the EPU software (ThermoFisher Scientific) and was monitored with cryoSPARC Live. A dataset consisting of 8402 movies was acquired with 0° stage tilt. A second dataset consisting of 4,288 movies was acquired with 30° stage tilt. Movies were acquired at ~6.8 e/Å^2^/s for a total exposure of ~42 e/Å^2^. The resulting movies were stored in the EER file format (57).

All cryoEM data were processed with cryoSPARC v4.4.0 (58). Initial patch-motion correction and patch-CTF estimation were carried out during data collection using cryoSPARC Live. Raw movie frames were grouped into 40 fractions with the upsampling factor set to 1 (i.e. no superresolution information used). Particle images were initially selected from a subset of the dataset using blob picker in cryoSPARC. After several rounds of 2D classification, a curated particle dataset was used for training a neural network model using the Topaz package (59). The Topaz model was then used for selecting particle images from all micrographs. Particle images were classified with iterative rounds of 2D classification and heterogenous refinement. Particle images of TcdB:cspg67 complexes were separated from the unbound TcdB particle images by 3D classification using a mask encompassing the cspg67 minibinder volume. The downstream cryo-EM refinements were carried out with the TcdB:cspg67 particle images only.

Reference-based motion correction and global CTF refinement were carried out to improve the resolution of the final map. The final nonuniform refinement map reached a resolution of 3.0 Å. The cryo-EM maps were deposited in the EMDB under accession number EMD-48223.

Initial atomic model building was carried out by docking an *apo* TcdB atomic model built in a previous cryo-EM study and originally derived from a predicted alphaFold model (Uniprot entry: P18177; Miletic et al., in preparation). A predicted model for cspg67 was docked into the TcdB:cspg67 complex cryo-EM map and combined with the TcdB model using ChimeraX (51). The model was then manually adjusted with ISOLDE (52) and refined with phenix.real_space_refine (60). The atomic model of the TcdB:cspg67 complex was deposited in the PDB with accession code 9MF4.

### Yeast Surface Display.

Both the initial design library and subsequent SSM libraries for the Frizzled-blocking binders (fzd library) and CSPG4-blocking binders (cspg library) were ordered as chip DNA oligos (Agilent). The initial library size was 15,000 and 2,811 for the fzd and cspg libraries, respectively. The fzd designs were flanked with 5′ GGTGGATCAGGAGGTTCG and 3′ GGAAGCGGTGGAAGTGGG adapters and cspg had 5′ TCGTCTGGTAGTTCAGGC and 3′ GGTTCTAGTGGCTCATCG adapters to enable subpool amplification. Libraries were qPCR amplified using Kapa hifi polymerase (Kapa Biosystems) in duplicate 25 μL reactions with the first reaction used to determine the cycle number to achieve half maximal amplification. Then a second production run was performed with these reactions purified from an agarose gel. The gel purified DNA fragments were amplified again to obtain sufficient material for electroporation in EBY100 with 2 μg of linearized petcon3 and 6ug of insert (61).

Yeast cultures were maintained in C-Trp-Ura media with 2% glucose (CTUG) at 30 °C. 18 h prior to sorting, 10 mL of SGCAA with 0.2% glucose was inoculated with 250 μL of C-Trp-Ura culture. In preparation for sorting, the cells were harvested at 4,000×*g* for 3 min followed by a wash step in PBS with 1% BSA (PBSF). Both yeast libraries and SSMs underwent four sorts. We began with an expression sort staining with only anti-myc FITC (Immunology Consultants Laboratory) followed by two rounds of “avidity sorts” where the cells were stained with both anti-myc FITC and streptavidin conjugated to phycoerythrin (SAPE, Thermo Fisher) mixed with target protein in a 1:4 ratio. For the fzd library, the biotinylated RBD toxin fragment was used while for the cspg library the biotinylated TcdB 1-2100 toxin was used. Finally we finished with a titration sort with streptavidin-PE conjugated to target protein in a 1:1 ratio to rank the binders by SC50, as described previously (16). For each sort, cells were collected then grown overnight in CTUG. 1 mL of culture was harvested and plasmid DNA extracted using a yeast plasmid miniprep kit (Zymo) and prepared for illumina sequencing through two rounds of PCR to attach pool specific barcodes. Reads were assembled using PEAR (62) and SC50s determined using a set of previously published computational tools from Cao et al (16). For combo libraries, no SC50s were calculated and rather the most represented designs at the lowest sort concentration were taken forward. For fzd designs, the top 94 based on abundance at 37 pM sorting concentration were taken forward for affinity screening. For cspg binders, due to the difficulty of working with full-length TcdB on surface based kinetic assays, the top 24 designs ranked by abundance at 20 pM were tested in neutralization assays. The titration series for the libraries are included in *SI Appendix*, Table S5.

### TcdB Expression.

Plasmid pHis1522 encoding his-tagged TcdB1 (VP10463) was a kind gift from Hanping Feng (University of Maryland Dental School, Baltimore, MD, 2120), and the plasmid for TcdB 027 in pHis1522 was synthesized by Genscript USA. TcdB was purified from *Bacillus megaterium* (MoBiTec, Germany) carrying the vector pHIS1522 encoding C*. difficile* TcdB VP10463 or 027 fused to a C-terminal His tag. From glycerol stocks, overnight starter cultures of *B. megaterium* were grown in lysogeny broth (LB) with tetracycline selection. The next morning, starter cultures were used to inoculate 1 to 2 L of terrific broth (TB) with tetracycline selection, and were grown at 37 °C, 180 RPM to an OD600 ≥ 0.8, then induced with 0.5% (w/v) xylose overnight at 30 °C, 180 RPM. The following morning, cells were pelleted at 4,000×*g* for 12 min and pellets were resuspended in lysis buffer containing 20 mM Tris pH8, 0.1 M NaCl, 1 mg/ml lysozyme, 1% v/v protease inhibitor cocktail P8849 (MilliporeSigma), and 100 U/ml Pierce universal nuclease (88701, ThermoFisher). The suspension was then passed twice through an EmulsiFlex C3 microfluidizer (Avestin) at 15,000 psi to lyse the cells. Cell lysate was clarified by centrifugation at 14,000×*g*, 4 °C for 20 min and supernatants were filtered with a 0.2 μM filter prior to running on a 5 mL HisTrap FF Ni-NTA column using an Äkta fast protein liquid chromatography (FPLC) systems (Cytiva). Proteins were eluted using a gradient of buffer containing 500 mM imidazole and a single peak was collected, and then further purified on a 1 ml HiTrap Q column (Cytiva). Proteins were eluted using a gradient of buffer with 100 mM to 1 M NaCl. When required for cryoEM purposes, collected fractions containing TcdB were then run by size exclusion on a Superose 6 increase 10/300 GL (Cytiva) to further polish the protein preparation and to buffer exchange TcdB into 20 mM Tris, 50 mM NaCl pH 8.0. Purified protein was either directly used for cryoEM analysis, or flash frozen in liquid nitrogen with 10% glycerol and stored at −80 °C.

For yeast screening purposes, C-terminal truncated TcdB1 (amino acids 1−2,100) with inactivating W102A/D286N/D288N mutations in the glucose transferase domain was generated in the pHis1522 vector, and purified protein was isolated as described above. Biotinylation was performed by incubating purified TcdB with a 15-fold molar excess of maleimide biotin (ThermoFisher) overnight at 4 °C in 20 mM Tris pH7.5, 150 mM NaCl, 5% glycerol. The biotinylated TcdB was then purified on a 1 ml HiTrap Q column (Cytiva).

Alternatively, TcdB 1285-1804 with C-terminal Avitag (RBD toxin fragment) was generated and purified as above, followed by overnight biotinylation reaction at 4 °C using a BirA biotin labeling kit (Avidity), then desalting with a Zeba 40k MW biotin-removal spin column (ThermoFisher).

Successful biotinylation was verified by Western blot, using streptavidin horseradish peroxidase as the detection agent.

### *E. coli* Expression of Designed Proteins.

Designed proteins were ordered as eblock fragments (Integrated DNA Technologies) with flanking BsaI cut sites and then cloned into LM0627 (Addgene 191551) which encodes an N-terminal MSG and C-terminal SNAC (63) and 6xhis tag. An Echo acoustic liquid handler (Beckman Coulter) was used to dispense 1 μL reaction volumes (0.1 μL water, 0.1 μL T4 ligase buffer, 0.375 μL eblock fragment at 4 ng/μL, 0.06 μL of BsaI-HFv2, 0.1 μL T4 ligase, 0.275 μL destination vector at 50 ng/μL). These reactions were incubated at 37 °C for 20 min before inactivation at 60 °C for 5 min. 5 μL of BL21 competent cells (or, in the case of the disulfide stabilized cspg binders, T7 shuffle express) (NEB) were dispensed onto the 1 μL reactions, and then incubated on ice for 30 min before 10 s heat shock at 42 °C. After 1 h recovery at 37 °C in 100 μL SOC media, 4 × 1 mL cultures of autoinduction media were inoculated with 25 μL of transformed cells. These were grown for 20 h at 37 °C for BL21 and 30 h for T7 shuffle express.

Cells were harvested through centrifugation at 4,000×*g* for 5 min and then lysed with 100 μL of BPER supplemented with 0.1 mg/mL lysozyme, 10 μg/mL DNAse I and 1 mM PMSF. The pellets were incubated in lysis buffer for 15 min on a shaker at 1,000 RPM before pooling and clarifying the lysates via centrifugation at 4,000×*g* for 10 min. The supernatant was then applied to 50 μL of Ni-NTA resin (Thermo Fisher), washed 3× with 300 μL of wash buffer (20 mM Tris, 300 mM NaCl, 25 mM imidazole) and then eluted in 200 μL of elution buffer (20 mM Tris, 300 mM NaCl, 500 mM imidazole pH 8). Elutes were injected onto an S75 5-150 GL Increase column via autosampler and underwent size exclusion chromatography into PBS (or HBS-EP for SPR experiments as described below). Fractions were normalized to 10 μM using an OT2 (Opentrons). Aggregation states were determined by plotting the elution volume of each peak relative to a standard curve for that column and yields were determined via integration of the SEC elution curve adjusted for 4 mL culture volume.

### Surface Plasmon Resonance.

For all SPR experiments, the analyte proteins (that which is flowed over the chip) were purified in HBS-EP (0.01 M HEPES pH 7.4, 0.15 M NaCl, 3 mM EDTA, 0.005% v/v Surfactant P20) (Cytiva). All experiments were run at 25 °C. For both the Frizzled binding site of TcdB and the SEMA6A binding site of TcsL, approximately 500 units of biotinylated RBD of the respective toxin was captured onto a biotin capture chip (Cytiva). For the single cycle kinetic runs, the binders were injected at 30 μL/min for 120 s, followed by 60 s dissociation and injection of the next concentration for six steps. The concentration series used in each experiment is described in the associated figure legend. After the sixth step, the dissociation was measured for 10 min to > 1 h (as indicated in the figure axis). For cspg binders where the full-length toxin had to be used, the miniprotein was captured on a CM5 chip through amine conjugation (Cytiva). Then, the full-length toxin in HBS-EP was flowed over in a 6-step single cycle kinetics run, in the same fashion as for the RBD binders. All single cycle kinetic data are shown with the measured data in black and the global Langmuir 1:1 fitting in red. The fitting was done using the Biacore 8 K Evaluation software (Cytiva) and then replotted in seaborn.

### Simulated Intestinal Fluid Assay.

For both simulated intestinal fluid (0.1 mg/mL trypsin, 0.1 mg/mL chymotrypsin, 3 mM sodium taurocholate, 19 mM maleic acid, 34.8 mM NaOH, 68.6 mM NaCl and 0.2 mM lecithin pH 6.5), 25 μM of binder was incubated at 37 °C for 5, 10, or 60 min (0 min condition was without protease). The reaction was quenched through the addition of 1 mM PMSF followed by incubation at 95 °C for 5 min. These samples underwent SDS-PAGE using a Criterion TGX stain free gel (BioRad) at 200 V for 30 min before being stained with coomassie blue.

### TcdB Neutralization Assays.

Vero (ATCC) and Vero CSPG4-knockout cells (64) were grown in DMEM with 10% fetal bovine serum in the presence of penicillin and streptomycin, at 37 °C, 5% CO2.

For cell assays that test TcdB toxicity through both the FZD and CSPG4 receptors, Vero cells were seeded at 5,000/well in 96-well clear CellBind plates (Corning) and used 24 h post plating. Test miniproteins were serial diluted in PBS, then added using a Bravo liquid handler (Agilent), followed immediately by recombinant TcdB1 to a final concentration of 0.1 pM. Cell viability was assessed 48 h later, when >99% of the vehicle control cells appeared to be rounded, by adding alamarBlue (ThermoFisher) and reading fluorescence signal (ex:555; em:585) 3 h later in a Spectramax m5 plate reader (Molecular Devices).

Alternatively, for cell assays favoring TcdB entry through FZD receptors, Vero CSPG4-knockout cells were seeded in 96-well plates at 5,500 cells/well. Using a Bravo liquid handler (Agilent), serial diluted test samples were added to cells, immediately followed by TcdB1 to a final concentration of 1.5 to 3 pM. Viability was assayed by adding alamarBlue (Thermofisher) after microscopic examination indicated cell rounding in the vehicle control wells (up to 48 h post TcdB addition), and reading fluorescence signal (ex:555; em:585) 3 h later in a Spectramax m5 plate reader (Molecular Devices).

For testing cspg minibinders, recombinant TcdB2 was used because it binds preferentially to the CSPG4 receptor. Vero cells were seeded at 5,000/well in 96-well plates, and incubated overnight. Serial diluted samples were added to wells using a Bravo liquid handling robot (Agilent), immediately followed by TcdB 027 to a final concentration of 0.1 pM. Cell viability was assessed after 24 h by adding alamarBlue Cell Viability Reagent (aka Resazurin, ThermoFisher), when microscopic examination indicated cell intoxication (ie., cell rounding, apoptotic bodies) in the vehicle control cells, and reading fluorescence signal (ex:555; em:585) 3 h later in a Spectramax m5 plate reader (Molecular Devices).

Neutralization curves are a representative curve from independent replicates while the reported IC50s are the mean from across replicates with the 95% CI.

### Secretion of Minibinders from *S. boulardii*.

Minibinder fusions to the secretion signal of the Mat-alpha mating pheromone from *Saccharomyces cerevisiae* were expressed in a *S. boulardii* MYA-796 wild type strain using the *ADH2* constitutive promoter in a 2 micron plasmid vector. The probiotic genome was not further engineered to increase secretion levels in order to avoid potential impacts on yeast fitness and concomitant effects on yeast survival in the GI tract. In particular, while overexpression of protein disulfide isomerase can improve the secretion of proteins containing disulfide bonds, such as single-chain antibody fragments, we found that miniproteins with engineered disulfide bonds expressed well in a wild type context and did not compromise yeast fitness, consistent with their successful expression in BL21/T7 shuffle express.

*S. boulardii* cells were grown in liquid XY media (2% bactopeptone, 1% yeast extract, 0.01% adenine sulfate, 0.02% tryptophan), 200 ug/mL G418, 2% ethanol, 2% glycerol for 3 d at 30 °C. Cells were centrifuged and supernatants were pelleted by centrifugation and concentrated 10-fold with a speedvac (Vacufuge) at 45 °C. Proteins were separated by 20% acrylamide/2% bisacrylamide SDS−PAGE. Minibinders in the supernatant were quantified with a direct ELISA using the nanobody Sb68-FLAG as a reference for the standard curve. For ELISA, the primary FLAG antibody used was conjugated with HRP (Sigma, A8592-1MG).

### TcsL Neutralization Assays.

Neutralization of TcsL was tested using HCT116 and HULEC5a cells (ATCC). Cells were seeded at a density of 4,000 cells/well in 96-well clear CellBind plates (Corning). The next day following cell attachment, minibinders were serial diluted in PBS with indicated concentration of TcsL. The solution was then added to cells and incubated for 48 h at 37 °C, 5% CO2. Cell viability was determined using alamarBlue (ThermoFisher), and fluorescence was read after 2 h using a Spectramax m5 plate reader (Molecular Devices). Neutralization curves are a representative curve from three independent replicates while the reported IC50s are the mean from across the three replicates.

### M79 Fusions.

B4 was produced as both an N- and C-terminal fusion to the albumin binding domain M79 (30) with 3 different linkers, GS, (G4S)_2_ and (G4S)_3_. All versions of these fusions retained TcsL inhibition. The design taken forward was B4-GS-M79. The sequences of B4 and B4-M79 and in *SI Appendix*, Table S1.

### In Vivo Evaluation of Protection from TcsL Toxic Shock.

Animal husbandry, ethical handling of mice and all animal work were carried out according to guidelines approved by Canadian Council on Animal Care and under protocols approved by the Centre for Phenogenomics Animal Care Committee (28-0431H). Female C67/Bl6J mice, 8 wk of age, were intraperitoneally injected with TcsL with or without B4 (anti-TcsL minibinder) fused to M79 (albumin binding domain). Treatment-naive, littermate female mice were group-housed and randomly assigned to experimental groups. In brief, 15 ng of TcsL was mixed with a 1,000-fold molar excess of B4-M79 for 1 h at 4 °C on a rotator prior to injection. After intoxication, animals were monitored closely and moribund animals were killed. For experiments that included follow-up dosing at 2 h intervals, each subsequent dose was the same as the initial dose of B4-M79, which corresponded to 0.06 mg/kg. All *P* values determined by the log-rank test for survival curves.

For histology, lungs were harvested and fixed in 4% paraformaldehyde for 24 h and then transferred to 70% ethanol for storage. Tissues were subsequently processed and embedded in paraffin following standard procedures at the CFIBCR Histology/Microscopy Core, University Health Network, Toronto, Canada. Paraffin blocks were sectioned at 4 μm then deparaffinized and stained for H&E using a Leica Auto Stainer XL.

### Data Visualization.

Figures were produced using PyMol, ChimeraX (51), Seaborn (65), and biorender.com.

## Supplementary Material

Appendix 01 (PDF)

## Data Availability

Cryo-EM structures data have been deposited in EMDB, PDB [EMD-45739 (66), EMD-48223 (67), 9MF4 (68), and 9CM5 (69)]. All other data are included in the manuscript and/or *SI Appendix*.

## References

[1] A. Y. Guh , Trends in U.S. burden of *Clostridioides difficile* infection and outcomes. N. Engl. J. Med. **382**, 1320–1330 (2020). 32242357 10.1056/NEJMoa1910215 PMC7861882

[2] G. P. Carter , TcsL is an essential virulence factor in *Clostridium sordellii* ATCC 9714. Infect. Immun. **79**, 1025–1032 (2011). 21199912 10.1128/IAI.00968-10 PMC3067498

[3] M. J. Aldape, A. E. Bryant, D. L. Stevens, *Clostridium sordellii* infection: Epidemiology, clinical findings, and current perspectives on diagnosis and treatment. Clin. Infect. Dis. **43**, 1436–1446 (2006). 17083018 10.1086/508866

[4] P. Agrawal, R. Garg, Fulminant leukemoid reaction due to postpartum *Clostridium sordellii* infection. J. Glob. Infect. Dis. **4**, 209–211 (2012). 23326079 10.4103/0974-777X.103899 PMC3543541

[5] D. M. Aronoff, J. D. Ballard, *Clostridium sordellii* toxic shock syndrome. Lancet Infect. Dis. **9**, 725–726 (2009). 19926032 10.1016/S1473-3099(09)70303-2

[6] P. Chen , Structural basis for CSPG4 as a receptor for TcdB and a therapeutic target in *Clostridioides difficile* infection. Nat. Commun. **12**, 3748 (2021). 34145250 10.1038/s41467-021-23878-3 PMC8213806

[7] J. Luo , TFPI is a colonic crypt receptor for TcdB from hypervirulent clade 2 *C. difficile*. Cell **185**, 980–994.e15 (2022). 35303428 10.1016/j.cell.2022.02.010

[8] P. Chen , Structural basis for recognition of frizzled proteins by *Clostridium difficile* toxin B. Science **360**, 664–669 (2018). 29748286 10.1126/science.aar1999 PMC6231499

[9] H. Lee , Recognition of semaphorin proteins by *P. sordellii* lethal toxin reveals principles of receptor specificity in clostridial toxins. Cell **182**, 345–356.e16 (2020). 32589945 10.1016/j.cell.2020.06.005 PMC7316060

[10] A. M. Seekatz, V. B. Young, Clostridium difficile and the microbiota. J. Clin. Invest. **124**, 4182–4189 (2014). 25036699 10.1172/JCI72336 PMC4191019

[11] Mark H. Wilcox , Bezlotoxumab for prevention of recurrent *Clostridium difficile* infection. N. Engl. J. Med. **376**, 305–317 (2017). 28121498 10.1056/NEJMoa1602615

[12] John G. Bartlett, Bezlotoxumab — A new agent for *Clostridium difficile* infection. N. Engl. J. Med. **376**, 381–382 (2017). 28121509 10.1056/NEJMe1614726

[13] F. Fitzpatrick, N. Safdar, J. Prehn, S. Tschudin-Sutter, How can patients with *Clostridioides difficile* infection on concomitant antibiotic treatment be best managed?. Lancet Infect. Dis. **22**, e336–e340 (2022). 35617982 10.1016/S1473-3099(22)00274-2

[14] D. N. Gerding , Bezlotoxumab for prevention of recurrent *Clostridium difficile* infection in patients at increased risk for recurrence. Clin. Infect. Dis. **67**, 649–656 (2018). 29538686 10.1093/cid/ciy171 PMC6093994

[15] G. J. Rocklin , Global analysis of protein folding using massively parallel design, synthesis, and testing. Science **357**, 168–175 (2017). 28706065 10.1126/science.aan0693 PMC5568797

[16] L. Cao , Design of protein-binding proteins from the target structure alone. Nature **605**, 551–560 (2022). 35332283 10.1038/s41586-022-04654-9 PMC9117152

[17] A. Roy , De novo design of highly selective miniprotein inhibitors of integrins αvβ6 and αvβ8. Nat. Commun. **14**, 5660 (2023). 37704610 10.1038/s41467-023-41272-z PMC10500007

[18] L. Cao , De novo design of picomolar SARS-CoV-2 miniprotein inhibitors. Science **370**, 426–431 (2020). 32907861 10.1126/science.abd9909 PMC7857403

[19] Z. Pan , Functional analyses of epidemic *Clostridioides difficile* toxin B variants reveal their divergence in utilizing receptors and inducing pathology. PLoS Pathog. **17**, e1009197 (2021). 33507919 10.1371/journal.ppat.1009197 PMC7842947

[20] P. Chen , Structure of the full-length *Clostridium difficile* toxin B. Nat. Struct. Mol. Biol. **26**, 712–719 (2019). 31308519 10.1038/s41594-019-0268-0 PMC6684407

[21] Y. Zhou , Structural dynamics of the CROPs domain control stability and toxicity of Paeniclostridium sordellii lethal toxin. Nat. Commun. **14**, 8426 (2023). 38114525 10.1038/s41467-023-44169-z PMC10730571

[22] N. R. Bennett , Improving de novo protein binder design with deep learning. Nat. Commun. **14**, 2625 (2023). 37149653 10.1038/s41467-023-38328-5 PMC10163288

[23] J. Dauparas , Robust deep learning-based protein sequence design using ProteinMPNN. Science **378**, 49–56 (2022). 36108050 10.1126/science.add2187 PMC9997061

[24] J.-J. Liu , Metabolic engineering of probiotic *Saccharomyces boulardii*. Appl. Environ. Microbiol. **82**, 2280–2287 (2016). 26850302 10.1128/AEM.00057-16 PMC4959471

[25] K. Chen , A probiotic yeast-based immunotherapy against *Clostridioides difficile* infection. Sci. Transl. Med. **12**, eaax4905 (2020). 33115949 10.1126/scitranslmed.aax4905 PMC7692727

[26] Fischer Marc , Fatal toxic shock syndrome associated with *Clostridium sordellii* after medical abortion. N. Engl. J. Med. **353**, 2352–2360 (2005). 16319384 10.1056/NEJMoa051620

[27] S. Tian , Genome-wide CRISPR screen identifies Semaphorin 6A and 6B as receptors for *Paeniclostridium sordellii* toxin TcsL. Cell Host Microbe **27**, 782–792.e7 (2020). 32302524 10.1016/j.chom.2020.03.007 PMC7228847

[28] J. L. Watson , De novo design of protein structure and function with RFdiffusion. Nature **620**, 1089–1100 (2023). 37433327 10.1038/s41586-023-06415-8 PMC10468394

[29] B. Geny , Clostridium sordellii lethal toxin kills mice by inducing a major increase in lung vascular permeability. Am. J. Pathol. **170**, 1003–1017 (2007). 17322384 10.2353/ajpath.2007.060583 PMC1864880

[30] H. van Faassen , Serum albumin-binding VH hs with variable pH sensitivities enable tailored half-life extension of biologics. FASEB J. **34**, 8155–8171 (2020). 32342547 10.1096/fj.201903231R

[31] L. Cegelski, G. R. Marshall, G. R. Eldridge, S. J. Hultgren, The biology and future prospects of antivirulence therapies. Nat. Rev. Microbiol. **6**, 17–27 (2008). 18079741 10.1038/nrmicro1818 PMC2211378

[32] Daniel A. Leffler, J. Lamont, *Clostridium difficile* infection. N. Engl. J. Med. **372**, 1539–1548 (2015). 25875259 10.1056/NEJMra1403772

[33] J. Chen , Cost-effectiveness of bezlotoxumab and fidaxomicin for initial Clostridioides difficile infection. Clin. Microbiol. Infect. **27**, 1448–1454 (2021). 33878506 10.1016/j.cmi.2021.04.004 PMC9478885

[34] Reuters, Merck to Discontinue Drug for Bacterial Infection (Reuters, 2024).

[35] Neoleukin Inc, United States security and exchange commission form 10-K. (2023). https://www.annualreports.com/HostedData/AnnualReportArchive/n/NASDAQ_NLTX_2022.pdf (Accessed 1 September 2024).

[36] S. L. Kordus , Nanobodies against *C. difficile* TcdA and TcdB reveal unexpected neutralizing epitopes and provide a toolkit for toxin quantitation *in vivo*. PLoS Pathog. **19**, e1011496 (2023). 37871122 10.1371/journal.ppat.1011496 PMC10621975

[37] Z. Peng , Designed ankyrin repeat protein (DARPin) neutralizers of TcdB from *Clostridium difficile* ribotype 027. mSphere **4**, e00596–19 (2019). 31578248 10.1128/mSphere.00596-19 PMC6796971

[38] X. Lv , De novo design of mini-protein binders broadly neutralizing Clostridioides difficile toxin B variants. Nat. Commun. **15**, 8521 (2024). 39358329 10.1038/s41467-024-52582-1 PMC11447207

[39] M. Glögl , Target-conditioned diffusion generates potent TNFR superfamily antagonists and agonists. Science **386**, 1154–1161 (2024). 39636970 10.1126/science.adp1779 PMC12416549

[40] J. Schilling, J. Schöppe, A. Plückthun, From DARPins to LoopDARPins: Novel LoopDARPin design allows the selection of low picomolar binders in a single round of ribosome display. J. Mol. Biol. **426**, 691–721 (2014). 24513107 10.1016/j.jmb.2013.10.026

[41] M. Guzzetta, A. Williamson, S. Duong, *Clostridium sordellii* as an uncommon cause of fatal toxic shock syndrome in a postpartum 33-year-old Asian woman, and the need for antepartum screening for this *Clostridia* species in the general female population. Lab. Med. **47**, 251–254 (2016). 27371657 10.1093/labmed/lmw025 PMC4985774

[42] D. M. Aronoff, J. M. Marrazzo, Infections caused by *Clostridium perfringens* and *Paeniclostridium sordellii* after unsafe abortion. Lancet Infect. Dis. **23**, e48–e55 (2023). 36155670 10.1016/S1473-3099(22)00590-4

[43] A. Elkbuli , Survival from Clostridium toxic shock syndrome: Case report and review of the literature. Int. J. Surg. Case Rep. **50**, 64–67 (2018). 30081323 10.1016/j.ijscr.2018.07.020 PMC6083381

[44] D. Schneidman-Duhovny, Y. Inbar, R. Nussinov, H. J. Wolfson, Patchdock and Symmdock: Servers for rigid and symmetric docking. Nucleic Acids Res. **33**, W363–W367 (2005). 15980490 10.1093/nar/gki481 PMC1160241

[45] J. Dou , De novo design of a fluorescence-activating β-barrel. Nature **561**, 485–491 (2018). 30209393 10.1038/s41586-018-0509-0 PMC6275156

[46] S. Yao , De novo design and directed folding of disulfide-bridged peptide heterodimers. Nat. Commun. **13**, 1539 (2022). 35318337 10.1038/s41467-022-29210-x PMC8941120

[47] D. N. Mastronarde, SerialEM: A program for automated tilt series acquisition on Tecnai microscopes using prediction of specimen position. Microsc. Microanal. **9**, 1182–1183 (2003).

[48] J. L. Rubinstein, M. A. Brubaker, Alignment of cryo-EM movies of individual particles by optimization of image translations. J. Struct. Biol. **192**, 188–195 (2015). 26296328 10.1016/j.jsb.2015.08.007

[49] A. Punjani, H. Zhang, D. J. Fleet, Non-uniform refinement: Adaptive regularization improves single-particle cryo-EM reconstruction. Nat. Methods **17**, 1214–1221 (2020). 33257830 10.1038/s41592-020-00990-8

[50] R. Sanchez-Garcia , DeepEMhancer: A deep learning solution for cryo-EM volume post-processing. Commun. Biol. **4**, 874 (2021). 34267316 10.1038/s42003-021-02399-1 PMC8282847

[51] E. C. Meng , UCSF chimeraX: Tools for structure building and analysis. Protein Sci. **32**, e4792 (2023). 37774136 10.1002/pro.4792 PMC10588335

[52] T. I. Croll, ISOLDE: A physically realistic environment for model building into low-resolution electron-density maps. Acta Crystallogr. D Struct. Biol. **74**, 519–530 (2018). 29872003 10.1107/S2059798318002425 PMC6096486

[53] P. Emsley, B. Lohkamp, W. G. Scott, K. Cowtan, Features and development of coot. Acta Crystallogr. D Biol. Crystallogr. **66**, 486–501 (2010). 20383002 10.1107/S0907444910007493 PMC2852313

[54] D. Liebschner , Macromolecular structure determination using X-rays, neutrons and electrons: Recent developments in Phenix. Acta Crystallogr. D. Struct. Biol. **75**, 861–877 (2019). 31588918 10.1107/S2059798319011471 PMC6778852

[55] C. J. Williams , MolProbity: More and better reference data for improved all-atom structure validation. Protein Sci. **27**, 293–315 (2018). 29067766 10.1002/pro.3330 PMC5734394

[56] C. R. Marr, S. Benlekbir, J. L. Rubinstein, Fabrication of carbon films with ∼ 500nm holes for cryo-EM with a direct detector device. J. Struct. Biol. **185**, 42–47 (2014). 24269484 10.1016/j.jsb.2013.11.002

[57] H. Guo , Electron-event representation data enable efficient cryoEM file storage with full preservation of spatial and temporal resolution. IUCrJ **7**, 860–869 (2020). 10.1107/S205225252000929X PMC7467176 32939278

[58] A. Punjani, J. L. Rubinstein, D. J. Fleet, M. A. Brubaker, CryoSPARC: Algorithms for rapid unsupervised cryo-EM structure determination. Nat. Methods **14**, 290–296 (2017). 28165473 10.1038/nmeth.4169

[59] T. Bepler , Positive-unlabeled convolutional neural networks for particle picking in cryo-electron micrographs. Nat. Methods **16**, 1153–1160 (2019). 31591578 10.1038/s41592-019-0575-8 PMC6858545

[60] P. V. Afonine , Real-space refinement in PHENIX for cryo-EM and crystallography. Acta Crystallogr. D. Struct. Biol. **74**, 531–544 (2018). 29872004 10.1107/S2059798318006551 PMC6096492

[61] L. Benatuil, J. M. Perez, J. Belk, C.-M. Hsieh, An improved yeast transformation method for the generation of very large human antibody libraries. Protein Eng. Des. Sel. **23**, 155–159 (2010). 20130105 10.1093/protein/gzq002

[62] J. Zhang, K. Kobert, T. Flouri, A. Stamatakis, PEAR: A fast and accurate Illumina paired-end read merger. Bioinformatics **30**, 614–620 (2014). 24142950 10.1093/bioinformatics/btt593 PMC3933873

[63] B. Dang , SNAC-tag for sequence-specific chemical protein cleavage. Nat. Methods **16**, 319–322 (2019). 30923372 10.1038/s41592-019-0357-3 PMC6443254

[64] P. Gupta , Functional defects in *Clostridium difficile* TcdB toxin uptake identify CSPG4 receptor-binding determinants. J. Biol. Chem. **292**, 17290–17301 (2017). 28842504 10.1074/jbc.M117.806687 PMC5655507

[65] M. Waskom, Seaborn: Statistical data visualization. J. Open Source Softw. **6**, 3021 (2021).

[66] C. Weidle, K. D. Carr, A. J. Borst, CryoEM strucuture of TcdB in complex with de novo minibinder fzd48. Electron Microscopy Data Bank. https://www.ebi.ac.uk/emdb/EMD-45739. Deposited 12 July 2024.

[67] S. Miletic, Z. Li, R. J. Ragotte, R. Melnyk, De novo designed minibinder complexed with Clostridioides difficile Toxin B. https://www.ebi.ac.uk/emdb/EMD-48223. Electron Microscopy Data Bank. Deposited 9 December 2024.

[68] S. Miletic, Z. Li, R. J. Ragotte, R. Melnyk, De novo designed minibinder complexed with Clostridioides difficile Toxin B. https://www.rcsb.org/structure/9MF4. Protein Data Bank. Deposited 9 December 2024.

[69] C. Weidle, K. D. Carr, A. J. Borst, CryoEM Strucuture of TcdB in complex with de novo minibinder fzd48. https://www.rcsb.org/structure/9CM5. Protein Data Bank. Deposited 12 July 2024.

