# Supplementary material for: De novo design of potent inhibitors of clostridial family toxins
Source: Proc Natl Acad Sci U S A. 2025 Sep 22;122(39):e2509329122. doi: 10.1073/pnas.2509329122 (PMC12501149; doi:10.1073/pnas.2509329122)
Supplement: Supplementary file 1 — Appendix 01 (PDF) [file pnas.2509329122.sapp.pdf]

Supplementary Tables

Table S1: Descriptions and amino acid sequences of key designs described in the paper. All designs were expressed with an N-terminal MSG- and C-terminal -GS - SNAC tag - 6x his tag, as described in the methods.

Name	Target	Description	Sequence
Group 1 frz parent	TcdB Frizzled site	Initial hit from frz group 1. All group 1 designs are sequence variants of this parent.	AEKEVQELIDKALNTPDPAV<br>ASKYIISAAYVAAKAKDPSL<br>LQKALDAAKKL
Group 2 frz parent	TcdB Frizzled site	Initial hit from frz group 2. All group 2 designs are sequence variants of this parent.	KEELEKMIEELKKLIEKGDK<br>EEFIKLLDEAFKKAKEAKDP<br>ETIAEVWKLSLEFKNKN
Group 3 frz parent	TcdB Frizzled site	Initial hit from frz group 3. All group 3 designs are sequence variants of this parent.	GEKGKLELLCKMLLTAAKLT<br>GKQKYKDALKELIEYLNEKG<br>YPDLAKEASAKLEEI
fzd5	TcdB Frizzled site	Sequence optimized group 1 design (post combo library)	AEKEVQELIDKALNTPDPAV<br>ASKYIISAVYVAAKARDPSL<br>FQKALDNAKKL
fzd13	TcdB Frizzled site	Sequence optimized group 2 design (post combo library)	AEKEVQELIDKALNTPDPKV<br>ASKYIISAVYVAAKARDPSL<br>FQSALDTAKKL
fzd24	TcdB Frizzled site	Sequence optimized group 3 design (post combo library)	GEKSKLELLCKMLLTKAKLT<br>GKQKYKNDLKELIEYLNEKG<br>YPDLAKEASAKLEEI
fzd48	TcdB Frizzled site	Sequence optimized group 2 design (post combo library)	KEELEKMIEELKKLIENGDK<br>ENFIKLFDEAFKKAKESRDP<br>RTIASVWTLVLEFKNKN
fzd84	TcdB Frizzled site	Sequence optimized group 2 design (post combo library)	KEELEKMIEELKKLIEKGDK<br>EKFIKLFDEAFKKAKESRDP<br>NTIAKVWKLVLEFKNKN
Group 1 cspg parent	TcdB CSPG4 site	Initial hit from cspg group 1. All cspg group 1 designs are sequence variants of this parent.	EEKLARAVVAVNKSSLATQL<br>FVRLYRLAKAGDPRAEEVAE<br>RLRKVLEMQGVPEEDIELVV<br>EVAKG
Group 2 cspg parent	TcdB CSPG4 site	Initial hit from cspg group 2. All cspg group 2 designs are sequence variants of this parent.	MDELVEKLKELLEKLKEKGD<br>EKGVKDTEKAIRICERSRHK<br>EFAEWAIEKAEARIKES
cspg4	TcdB CSPG4 site	Sequence optimized group 1 design (post combo library)	ENKLARAVVTVNKSWLATQL<br>FVRLYRLCKAGDPRCEEVAE<br>KLRKVLELQGVPETDIELVV<br>EVAKG
cspg18	TcdB CSPG4 site	Sequence optimized group 2 design (post combo library)	MDEKVEKLKECLEKLKEKGD<br>IKGVIDTERAIRIIERSRHK<br>EFAEWAIEKCENRIKES

cspg27	TcdB CSPG4 site	Sequence optimized group 2 design (post combo library)	MDELVEKLKECLEKLKEKGD IKGVTDTEKAIRIIERSRHK EFAEWAIEACEARIKES
cspg35	TcdB CSPG4 site	Sequence optimized group 2 design (post combo library)	MDELVEKLKECLEKLKEKGD LKGVKDTERAIRIMERSRHK EFAEWAIEKCEARIKES
ss2cspg18	TcdB CSPG4 site	Sequence optimized group 2 design (post combo library) (improved protease stability)	MDEKVEKLKECLEKLKEKGD IKGVIDTERAIRICERSRHK EFCEWAIEKCENRIKES
ss2cspg27	TcdB CSPG4 site	Sequence optimized group 2 design (post combo library) (improved protease stability)	MDELVEKLKECLEKLKEKGD IKGVTDTEKAIRICERSRHK EFCEWAIEACEARIKES
ss2cspg35	TcdB CSPG4 site	Sequence optimized group 2 design (post combo library) (improved protease stability)	MDELVEKLKECLEKLKEKGD LKGVKDTERAIRICERSRHK EFCEWAIEKCEARIKES
cspg67	TcdB CSPG4 site	Sequence optimized group 2 design for which the cryoEM structure was solved	AEELAKKLEEMAEVFKEKGD EEGAKECLKAAKIVRRSKNE EFGKWCIKKAEERLKKL
F4	TcsL SEMA6A site	Initial hit for SEMA6A blockers	NEELIKEIKKLLKEGKPASE ILELLKKAGKEEIAKEAEKA ALSSYDMTKAAAEEAIKKLK
B4	TcsL SEMA6A site	ProteinMPNN redesign optimization of F4	TEEIIKEIKKMKEKGEKASS IIDLLKKAGYSEIAKKAELA ALKSENMTEAAAEKAIEELK
B10	TcsL SEMA6A site	ProteinMPNN redesign optimization of F4	NEEIIKEIKQLLKEGKKASE IIELLKKNGYEEIAKDAELA ALKSKDMRKAAAETAIKKLE
B4-M79	TcsL SEMA6A site	ProteinMPNN redesign optimization of F4 with half life extension, used in the in vivo studies	TEEIIKEIKKMKEKGEKASS IIDLLKKAGYSEIAKKAELA ALKSENMTEAAAEKAIEELK GSQVKLEESGGGLVQAGGSL KLSCAASGSTFSSSSVGWYR QAPGQQRELVAAITSGGSTN TADSVKGRFTMSRDNAKNTV YLQMRDLKPEDTAVYYCNVA GRNWVPISRYSPGPYWGQGT QVTVSS

Table S2: Kinetic parameters for surface plasmon resonance experiments of designed miniproteins against both TcdB sites and TcsL.

Design	Group	Target	Antigen used	Figure Reference	k_{on} ($M^{-1}s^{-1}$) (standard error)	k_{off} (s^{-1}) (standard error)	KD (M)
fzd13	fzd group 1	TcdB (Frizzled site)	TcdB RBD	Figure 2B	2.53E7 (1.99E5)	1.4E-4 (6.79E-7)	5.55E-12
fzd48	fzd group 2	TcdB (Frizzled site)	TcdB RBD	Figure 2B	3.42E7 (1.11E5)	3.33E-7* (2.24E-9)	9.75E-15*
fzd24	fzd group 3	TcdB (Frizzled site)	TcdB RBD	Figure 2B	4.24E7 (2.19E5)	6.49E-4 (1.55E-6)	1.53E-11
ssfzd84	fzd group 2	TcdB (Frizzled site)	TcdB FL	Figure S5	9.26E5 (8.94E1)	2.67E-5 (5.63E-8)	2.89E-10
cspg group 1 parent	cspg group 1	TcdB (CSPG4 site)	TcdB FL	Figure S8	3.1E6 (1.19E5)	5.88E-2 (1.70E-3)	1.89E-8
cspg group 2 parent	cspg group 2	TcdB (CSPG4 site)	TcdB FL	Figure S8	3.18E6 (1.63E4)	3.57E-2 (1.27E-4)	1.12E-8
cspg4**	cspg group 1	TcdB (CSPG4 site)	TcdB FL	Figure 3B	1.91E7 (5.07E5)	3.78E-2 (1.01E-3)	1.98E-9
ss2cspg18	cspg group 2	TcdB (CSPG4 site)	TcdB FL	Figure 3B	1.9E6 (2.19E3)	1.64E-3 (1.91E-6)	8.65E-10
ss2cspg27	cspg group 2	TcdB (CSPG4 site)	TcdB FL	Figure S8	1.99E5 (3.16E2)	1.01E-4 (1.45E-7)	5.07E-10
ss2cspg35	cspg group 2	TcdB (CSPG4 site)	TcdB FL	Figure S8	1.67E5 (2.20E2)	8.03E-5 (1.13E-7)	4.80E-10
cspg67	cspg group 2	TcdB (CSPG4 site)	TcdB FL	Figure S7	1.99E6 (2.66E3)	3.87E-3 (4.98E-6)	1.95E-9
F4	N/A	TcsL (SEMA6 A site)	TcsL RBD	Figure S10	2.17E6 (5.59E3)	9.28E-3 (1.91E-5)	4.28E-9
B4	N/A	TcsL (SEMA6 A site)	TcsL RBD	Figure 5	1.98E6 (1.31E3)	1.12E-4 (6.1E-8)	5.69E-11
B10	N/A	TcsL	TcsL RBD	Figure 5	6.86E6	1.25E-4	1.83E-11

		(SEMA6 A site)			(1.41E3)	(2.59E-8)	

*values beyond instrumentation limits and thus unlikely to be accurate
**this is not to be confused with CSPG4, the receptor that binds that site

Table S3: Cryo-EM data collection, refinement, and validation statistics for TcdB with CSPG4- and Frizzled-blocking minibinder.

**CryoEM data collection**	**TcdB:cspg67**	**TcdB:fzd48**
Magnification	75000	105,000
Voltage (kV)	300	300
Electron exposure	~42 e/Å2	47 e/Å2
Pixel size	1.03 Å	2.7 Å
Symmetry	C1	C1
Number of particles	179414	108,076
Map resolution	3.02 Å	4.64 Å
FSC threshold	0.143	0.143
**Model Composition**	**chain A: TcdB**	**chain A:TcdB**
Nonhydrogen atoms	8886	9,354
Protein residues	1095	1870
Modeled residues of TcdB	1-838, 1633-1805, 1811-1894	1-1094, 1196-1230, 1280-1352, 1373-1432, 1453-1986, 1997-2024
Starting model	Alphafold (Uniprot entry: P18177)	PDB ID: 60Q5
**Model Composition**	**chain B:cspg67**	**chain B:fzd48**
Nonhydrogen atoms	462	287
Protein residues	57	57
**Bonds (RMSD)**		
Bond Length (Å)	0.012	0.010
Bond Angle (°)	1.135	2.165
**Validation**		
MolProbity score	1.12	1.35
Clashscore	3.24	0.14
Poor rotamers	2	0
**Ramachandran plot**		
Favored (%)	98.34	75.56
Allowed (%)	1.66	19.31
Disallowed (%)	0	5.13

Table S4: Ultramer sequences for assembly of the combo libraries for fzd and cspg designs

Library	Ultramer 1	Ultramer 2
fzd group 1	TCGTCTGGTAGTTCAGGCGCAGAAAMAGAAGTA<br>CAAGAACTAATAGACAMAGCACTAAACACACCA<br>GACCCADHGGTAGCAAGCAAATACATAATAAGC<br>GCAKYATMCGTAGCAGCAMAAGCADVAGACCCA<br>AGCCTABWCCAAWMAGCADYAGACRHMGCAAAA<br>AAACTAGGTTCTAGTGGCTCATCG	
fzd group 2	TCGTCTGGTAGTTCAGGCAAAGAAGAACTAGAA<br>AAAATGATAGAAGAACTAAAAAAACTAATAGAA<br>RAMGGAGACMAAGAARMMTTCATAAAACTATTM<br>GACGAAGCATTCAAAAAA	CGATGAGCCACTAGAACCGTTTTTGTTTTTGAAT<br>TCTAGGVMTAGCNYCCATACTKHTGCTATTGTKY<br>BTGGGTCTBHTGMTTCTTTTGCTTTTTTGAATGC<br>TTCGTC
fzd group 3	TCGTCTGGTAGTTCAGGCRKAGAAMAARGCAAA<br>CTAGAACTACTADBCAAAATGCTACTAACARVA<br>GCAAAAMTGACAGGAAAMCAAAAATACAAARAC<br>RMCCTAAAAGAACTAATARAAHVMCTAAACGAA<br>DMAGGATACCCAGACCTAGCA	CGATGAGCCACTAGAACCTATTTCTTCTAGTTTT<br>GCGCTTGCTTCTTTTGCTAGGTCTGGGTATCC
cspg group 1	GGTGGATCAGGAGGTTCGGAARAMAAACTAGCA<br>AGAGCAGTAGTARCAGTAAACAAARRCTSGCTA<br>GCAACASWACTATTCGTAAGACTATACAGACTA<br>GCAAAAGCAGGAGACCCAAGAGCA	GCAGGAGACCCAAGAGCAGAAGAAGTAGCAGAAA<br>RMCTAAGAWRGGTAMYGGAAMTGCAAGGAGTAMC<br>AGAARMAGACATAGAACTAGTAGTAGAAGTAGCA<br>AAAGGAGGAAGCGGTGGAAGTGGG
cspg group 2	GGTGGATCAGGAGGTTCGATGGACGAAVWAGTA<br>GAAAAACTAAAAGAACTACTAGAAAAACTAAAA<br>GAAAAAGGAGACRHAAAAGGAGTARHAGACACA<br>GAAARAGCAATAMBAATATGCGAAAGAAGCAGA	ATATGCGAAAGAAGCAGAYHCAAAGAATTCGCAG<br>AATGGGCAATAGAARMAKCAGAARMCAGAATAAA<br>AGAAAGCGGAAGCGGTGGAAGTGGG

Table S5: DIlution series for yeast surface display FACS

Target site	Stage	Titration series
Frizzled	Initial	1000 nM, 100 nM, 10 nM
Frizzled	SSM	1000 nM, 100 nM, 10 nM, 1 nM
Frizzled	Combo	1 nM, 333 pM, 111 pM, 37 pM
CSPG4	Initial	1000 nM, 100 nM, 10 nM, 1 nM
CSPG4	SSM	1000 nM, 100 nM, 10 nM, 1 nM
CSPG4	Combo	1 nM, 100 pM, 20 pM

A B C

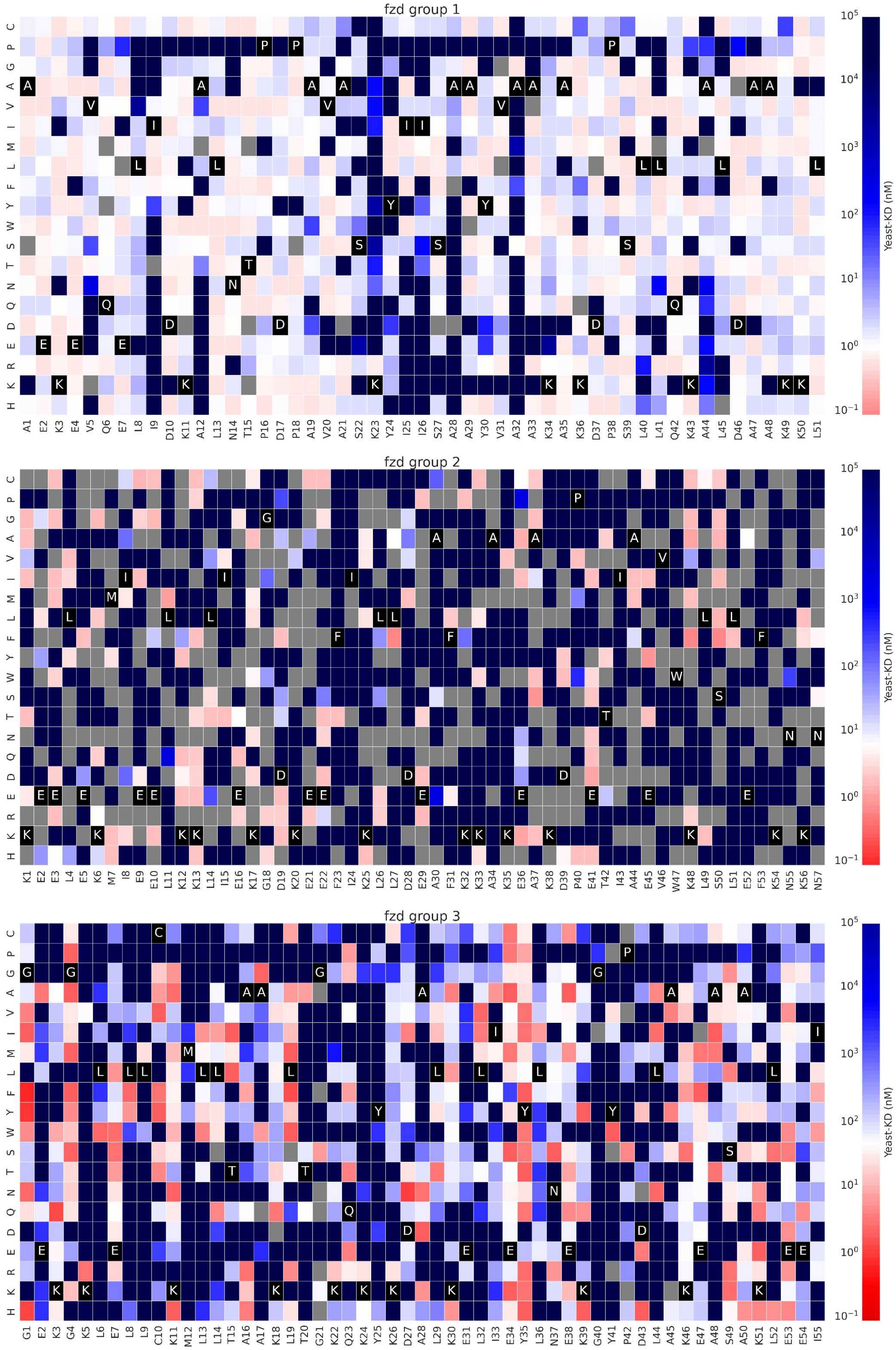

Figure S1. Yeast surface display SSM of group 1 (A), group 2 (B) and group 3 (C) fzd parental designs from which all the sequence optimized variants are derived. Yeast K_D is the SC50 as defined by Cao *et al.* (2022). Gray squares indicate that the variant was not identified in the library. Group 2 has poor library coverage, but was still taken forward as it was among the most promising initial hits based on preliminary neutralization data.

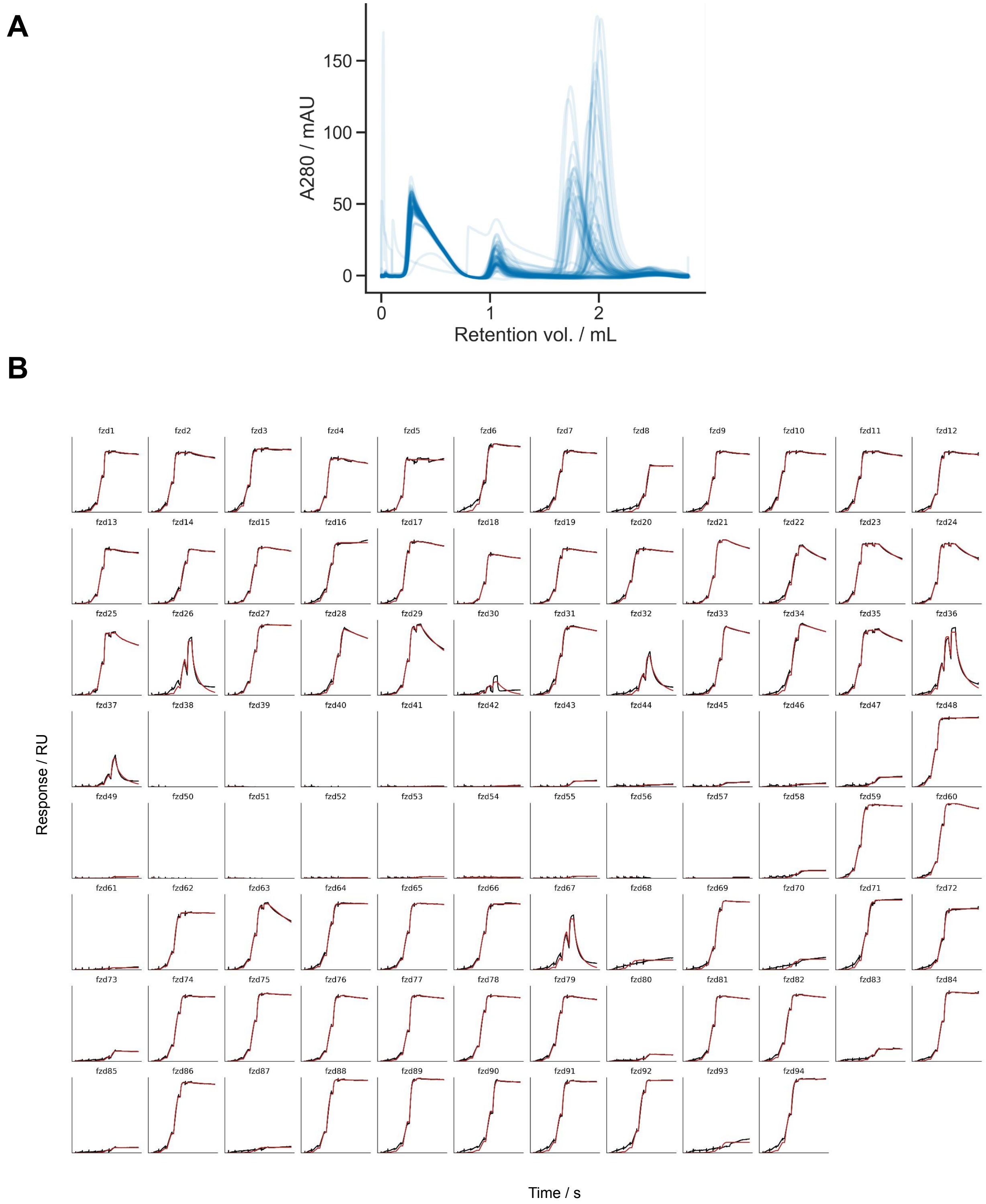

Figure S2. Optimization of Frizzled-blocking miniproteins. A. SEC traces of 94 designs sequence optimized designs from 4 mL culture. **B.** Affinity determination through SPR with the RBD of TcdB captured on the chip and a 6-step 5-fold dilution series of each miniprotein starting at 25 nM. Designs fzd38 to fzd47 and fzd49 - fzd58 failed to express, rather than failed to bind. Global fit is shown in red while the measured data is shown in black.

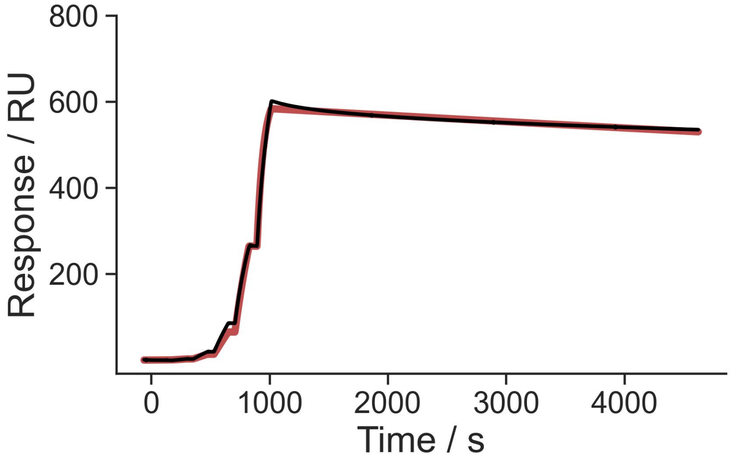

Figure S3. Binding of ssfzd84 to full length TcdB. ssfzd84 was immobilized via amine conjugation. Full length TcdB was used as an analyte over a 6-step 4-fold dilution starting at 100 nM.

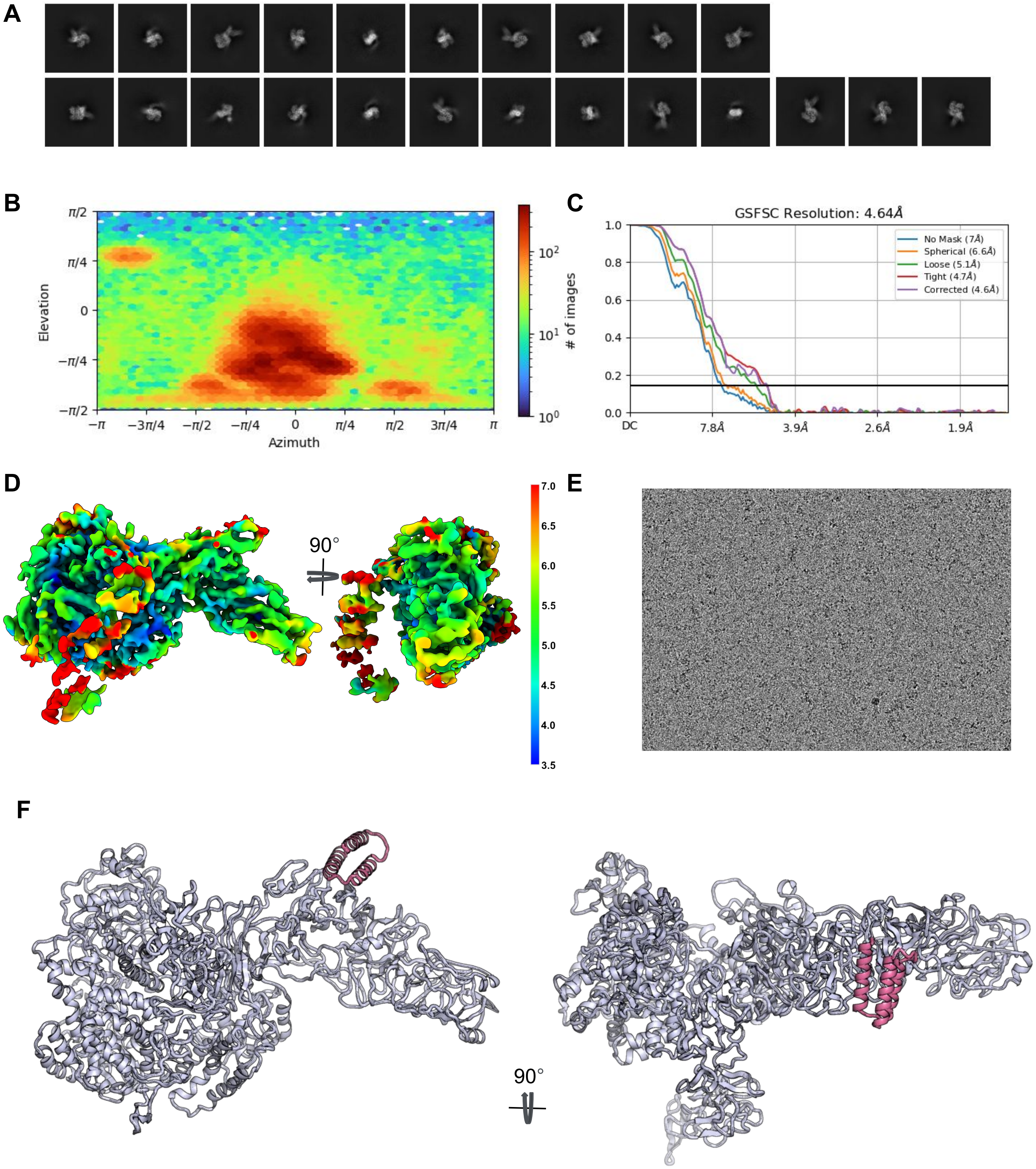

Figure S4. Determination of cryoEM structure of fzd48 bound to TcdB. A. Representative 2D class averages. **B.** Orientational distribution plot. **C.** Global Fourier Shell Correlation (FSC) following a gold standard refinement and with correction for the effects of masking. **D.** CryoEM density colored by resolution indicated by the scale bar (unit is Å). **E.** Representative micrograph. **F.** CryoEM model of fzd48 bound to TcdB.

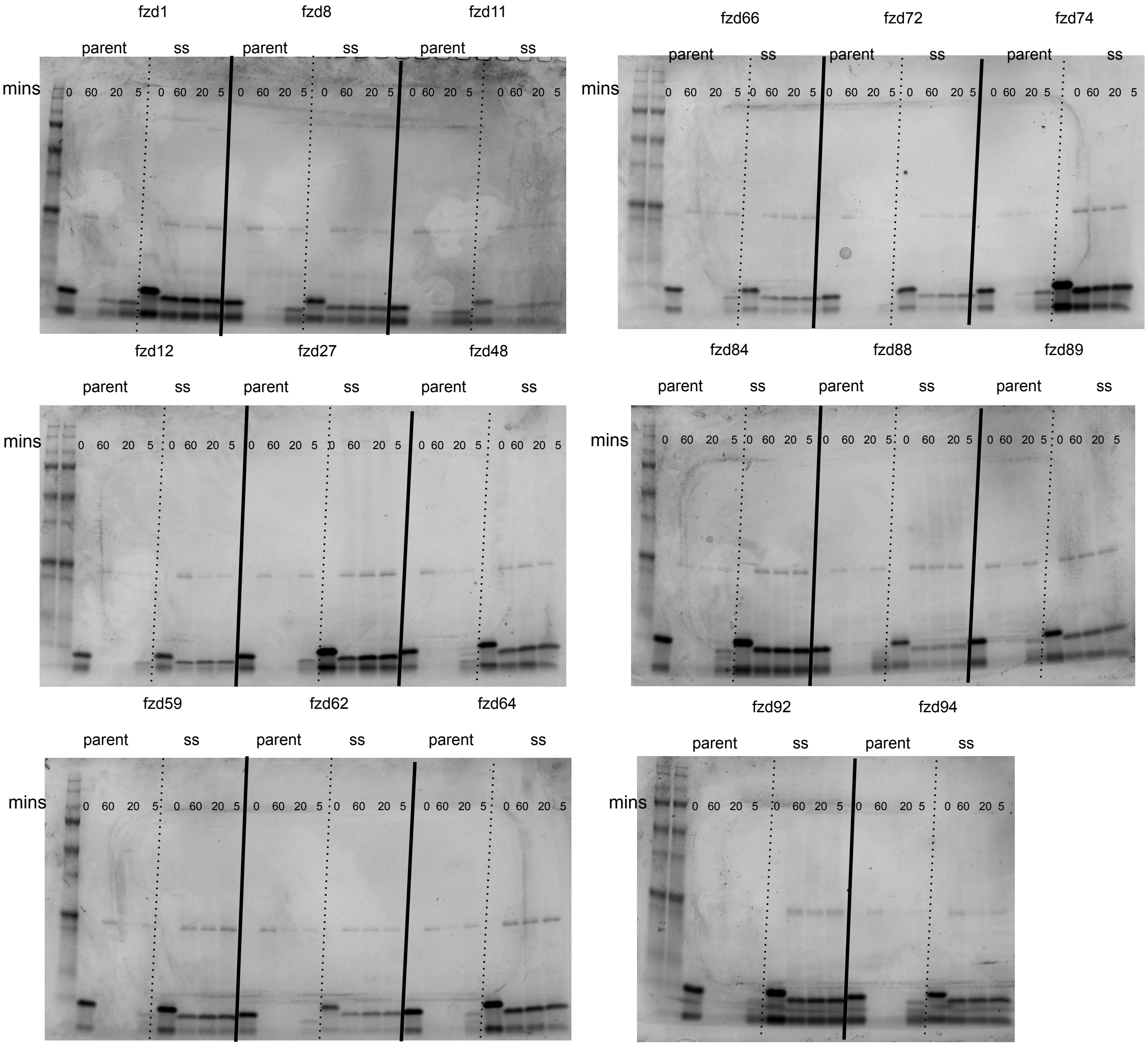

Figure S5. Disulfide stabilization of the Frizzled-blocking minibinders from group 1 and group 2. SIF assay where designs are incubated in simulated intestinal fluid for 5, 20 or 60 min. The 0 time point contains no protease. **"**Parent" is the design lacking disulfides while "ss" indicates the disulfide stabilized variant.

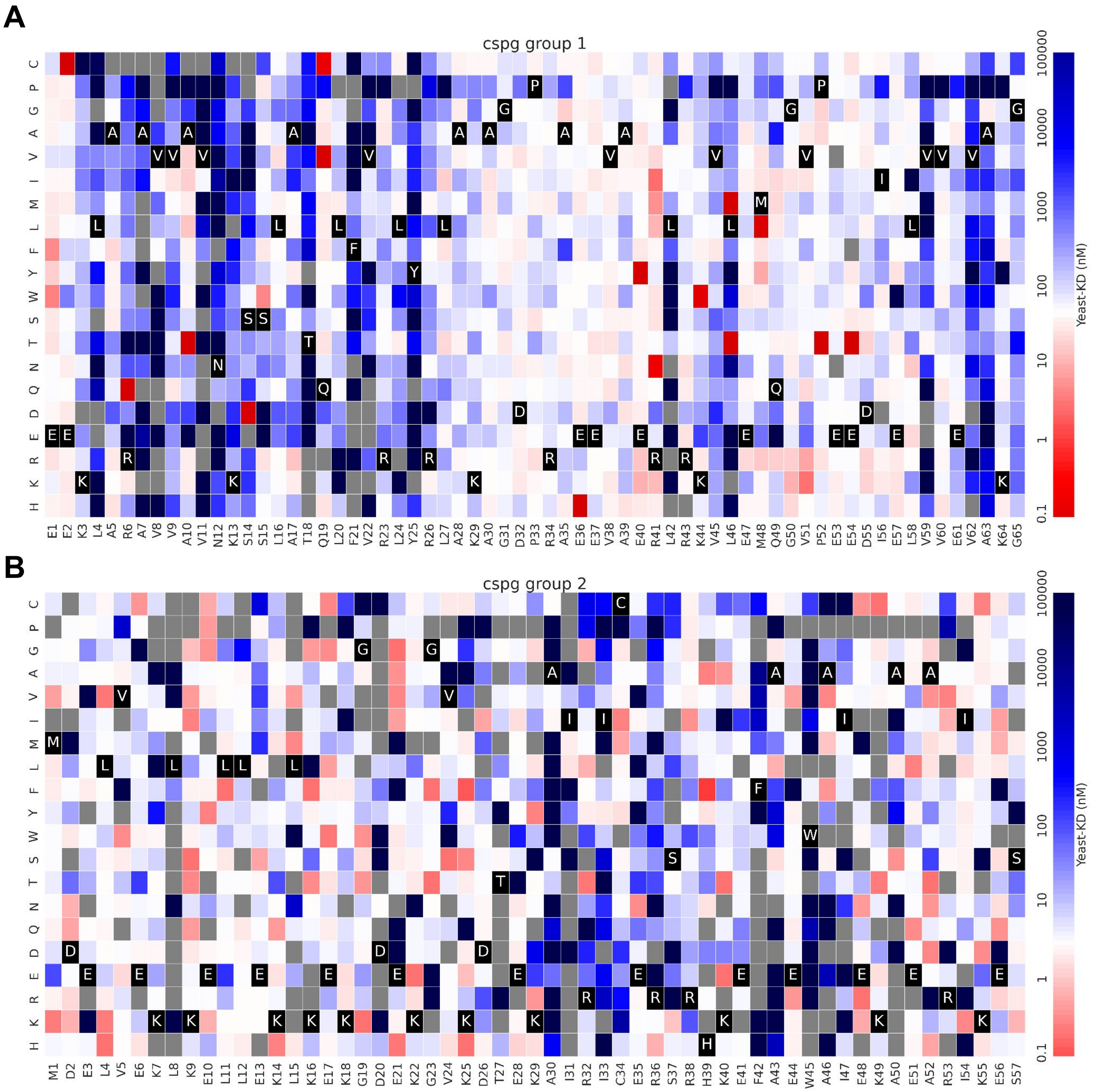

Figure S6. Yeast surface display SSM of group 1 (A) and group 2 (B) cspg parental designs from which all the sequence optimized variants are derived. Yeast K_D is the SC50 as defined by Cao *et al.* Gray squares indicate that the variant was not identified in the library.

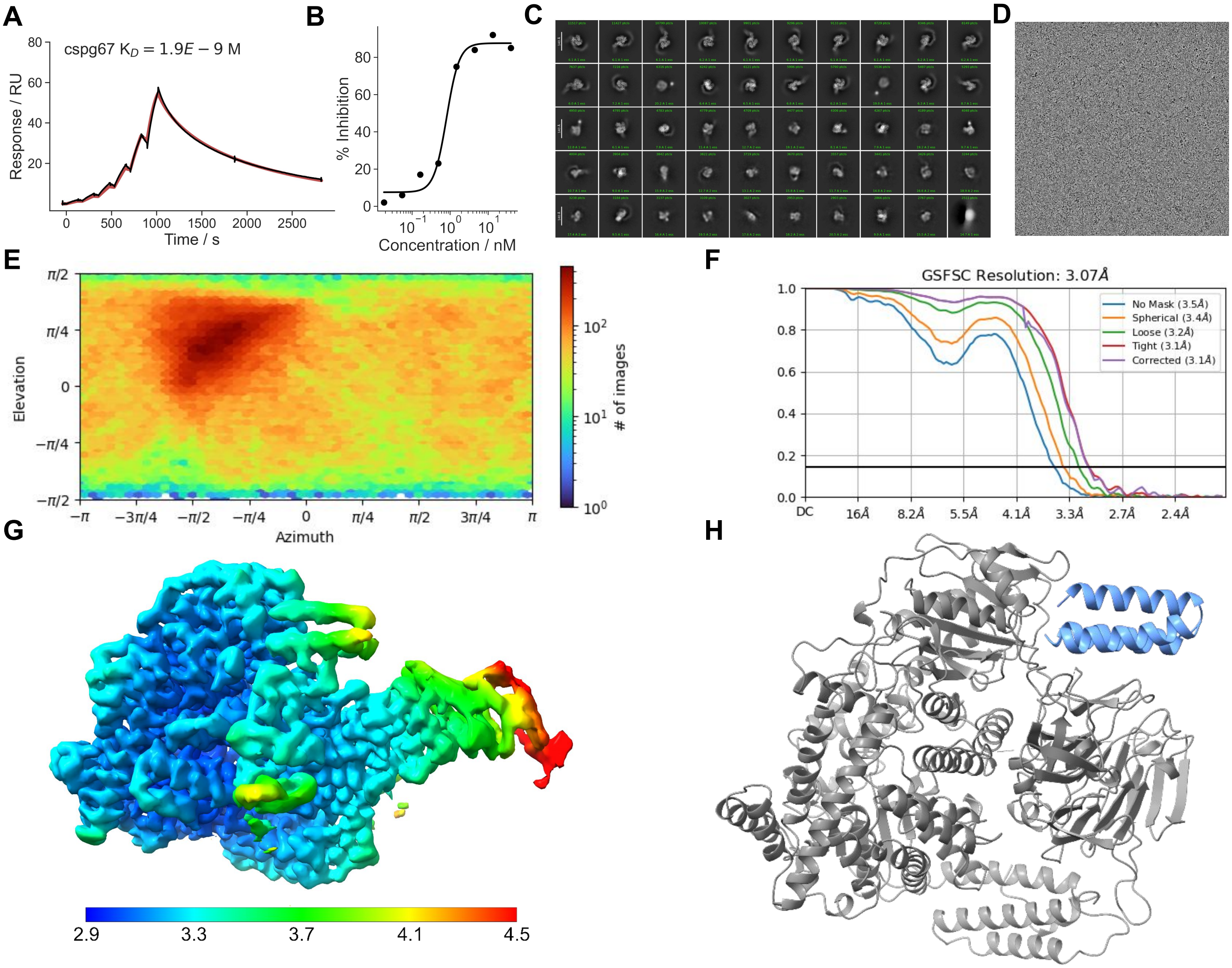

Figure S7. CryoEM structure of TcdB:cspg67. A. Single cycle kinetic analysis of cspg67 amine conjugated to a CM5 chip with full length TcdB used as the analyte. Injections began at 0.625 nM and then increased in concentration 2-fold with each subsequent injection across six steps to yield an upper concentration of 20 nM. **B.** Neutralization of TcdB2 in WT Vero cells by purified recombinant cspg67 for a single 8-step 3-fold dilution series starting at 40 nM. The IC50 was measured to be 1 nM (mean across 2 independent replicates) **C.** 2-D class averages **D.** Representative micrograph **E.** Orientational distribution plot. **F.** Global Fourier Shell Correlation (FSC) following a gold standard refinement and with correction for the effects of masking **G.** CryoEM density colored by resolution indicated by the scale bar (unit is Å) **H.** CryoEM model of cspg67 bound to TcdB.

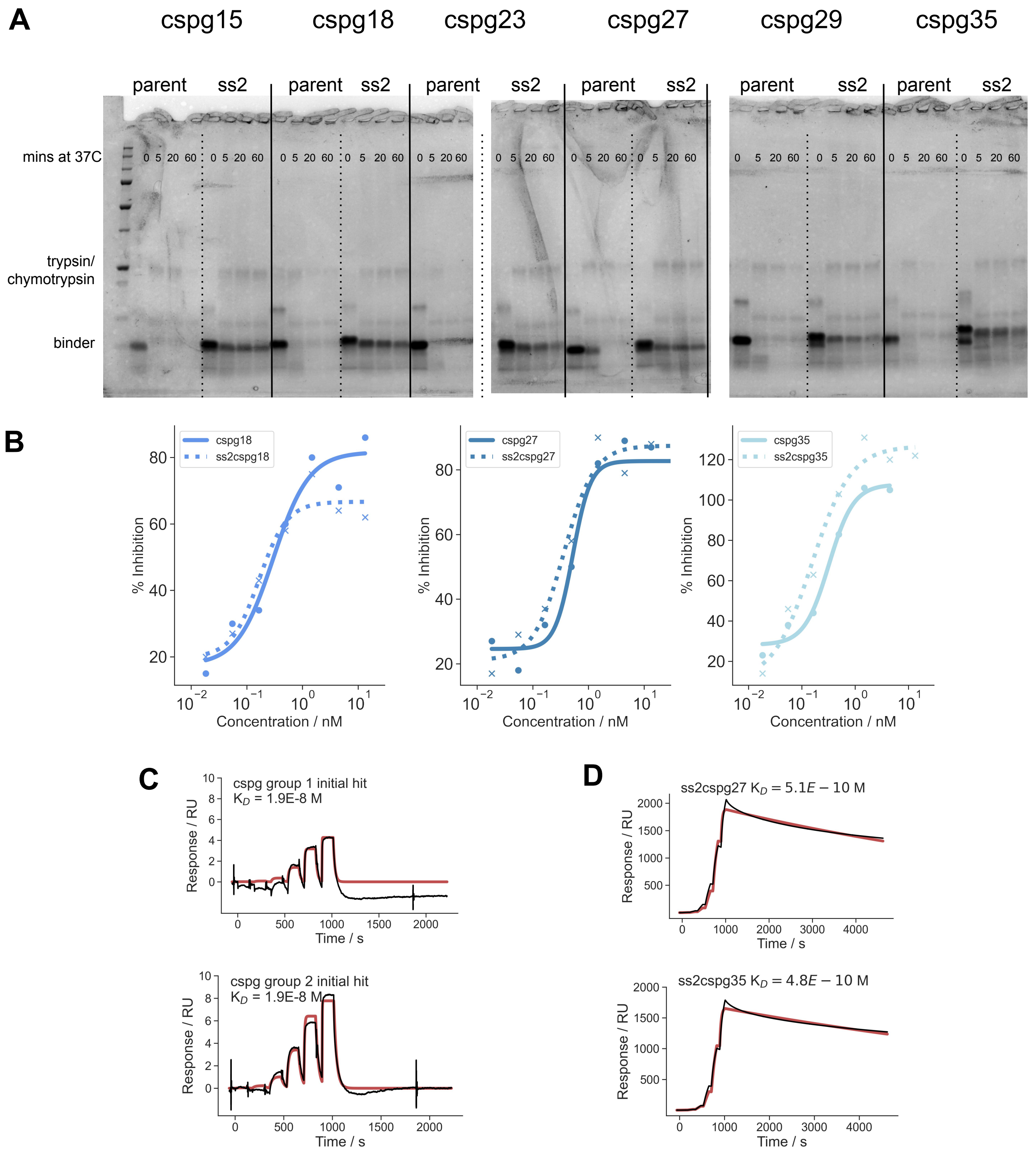

Figure S8. Introduction of two disulfide bonds to enhance protease stability of group 2 cspg designs. A. Time course incubation in simulated intestinal fluid at 37°C for the single disulfide parental designs (denoted parent) compared to the dual disulfide stabilized designs (denoted ss2). **B.** TcdB2 neutralization in WT Vero cells (CSPG4 dependent system) with 0.1 pM toxin comparing the parental designs (solid line with individual data points marked with •) to the dual disulfide stabilized designs (dashed lined with individual data points marked with x). IC50 values for each design are cspg18: 290 pM , ss2cspg18: 120 pM, cspg27: 540 pM, ss2cspg27: 270 pM, cspg35: 570 pM, ss2cspg35: 420 pM. **C.** Single cycle kinetic analysis of the initial hits (unoptimized, pre-SSM) for group 1 and group 2 cspg designs, from which the optimized hits are derived. The low overall response is due to large molecular weight difference between TcdB (captured on biotin CAP chip) and the minibinder, which is why the orientation was swapped for future experiments. Analysis used the minibinder as the analyte across a 5-fold, 6-step dilution series with an upper concentration of 200 nM **D.** Single cycle kinetic analysis of group 2 optimized designs ss2cspg27 and ss2cspg35 minibinder amine conjugated to the surface across a 4-fold, 6-step dilution series of full length TcdB with an upper concentration of 100 nM.

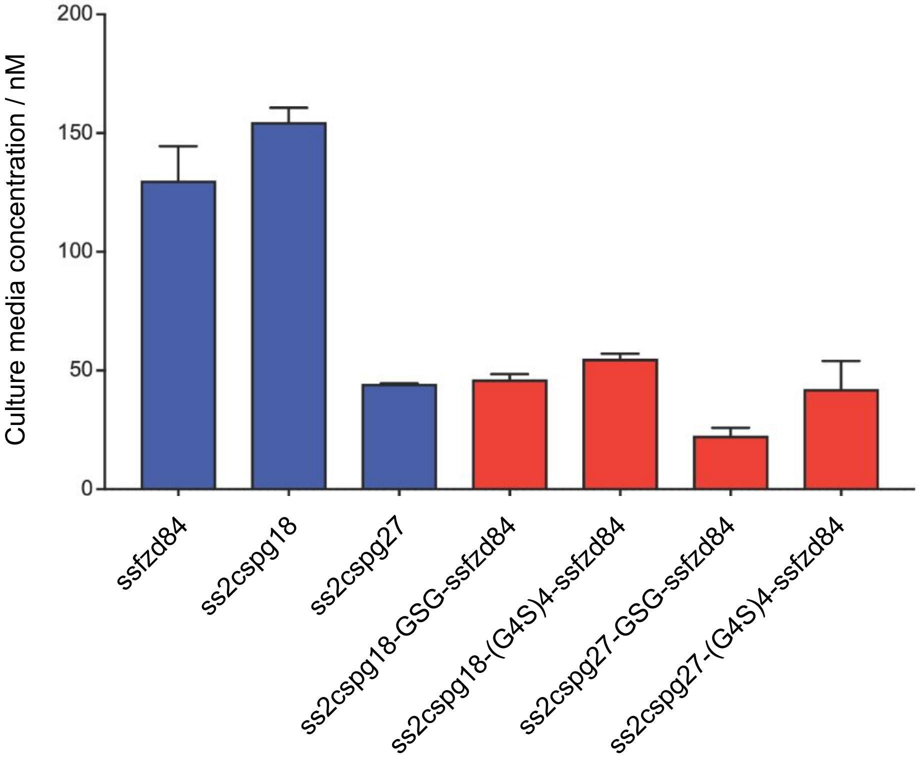

Figure S9. Secretion of fusion binder constructs from *S. boulardii.* A. Quantified secretion of monomeric ss2cspg designs and their fused counterparts to ssfzd84 into culture media.

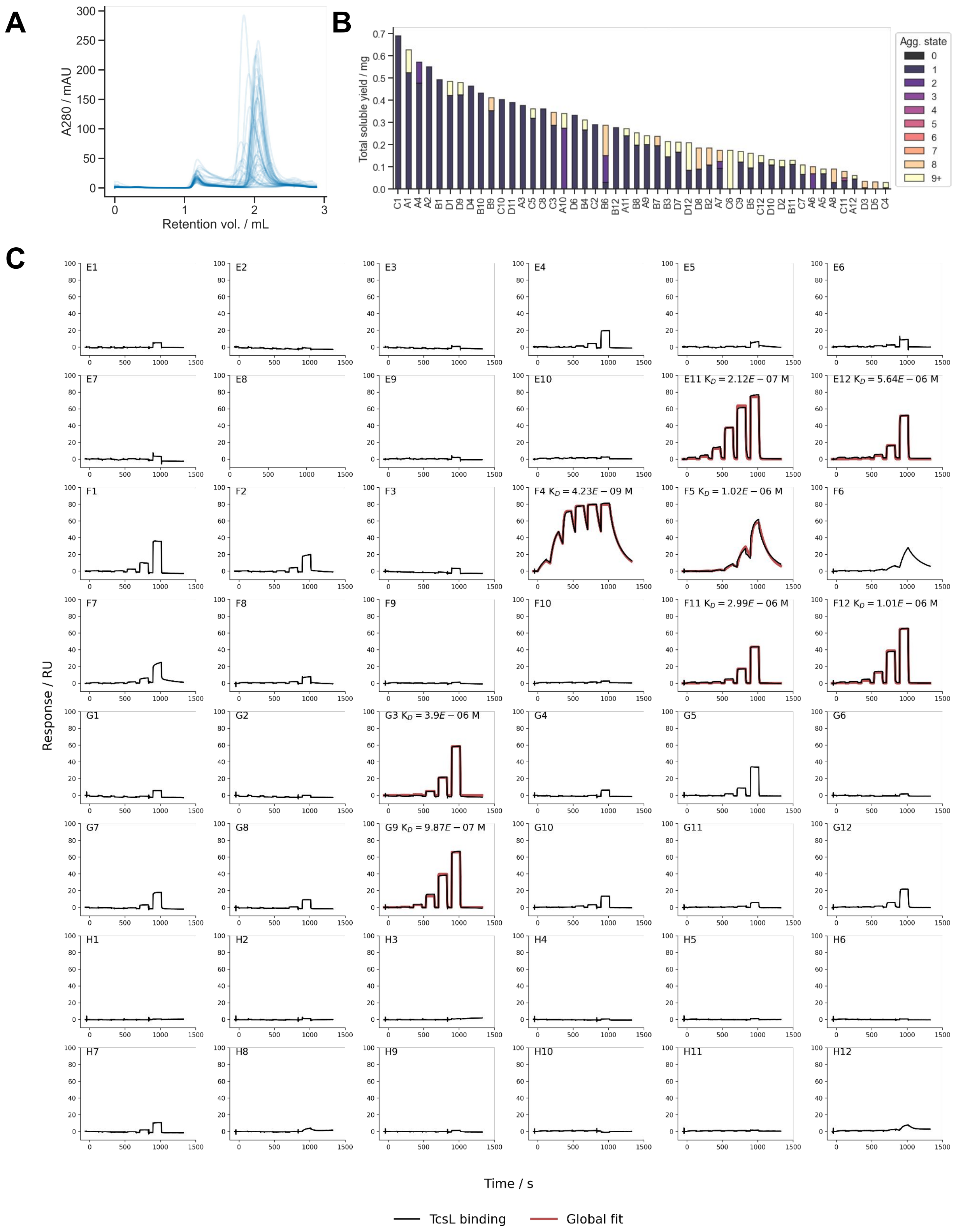

Figure S10. Characterization of SEMA6A-blocking miniproteins. A. SEC traces of all 48 designs from 4 mL cultures. **B.** Aggregation state(s) of each design based on SEC profile and molecular weight standard curve for the column. **C.** Affinity determination through SPR with the RBD of TcsL captured on the chip and a 6-step 5-fold dilution series of each miniprotein starting at 5000 nM. Global fit is shown in red while the measured data is shown in black.

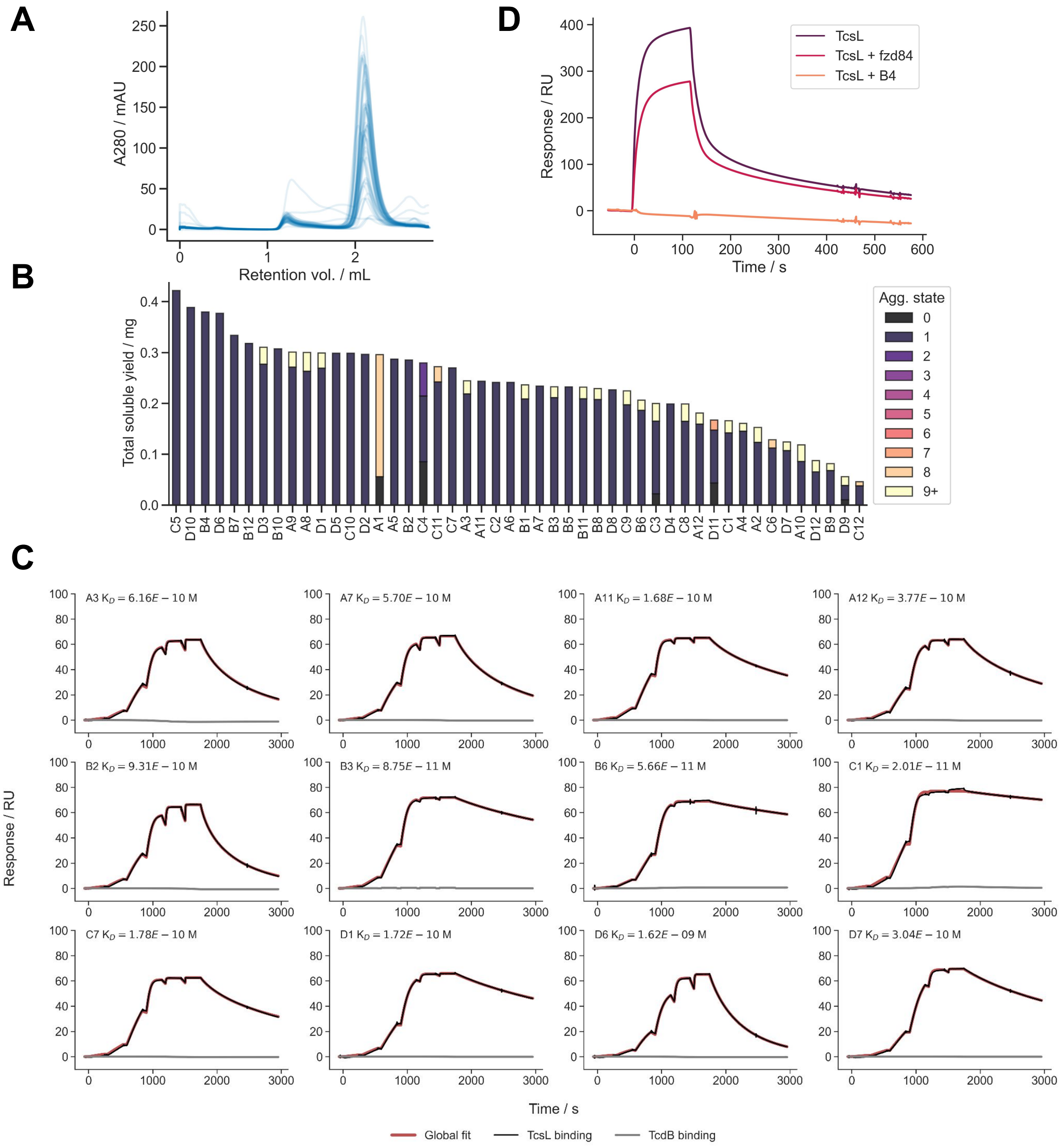

Figure S11. Optimization of SEMA6A-blocking miniproteins. A. SEC traces of all 48 designs from 4 mL culture. **B.** Aggregation state(s) of each design based on SEC profile and molecular weight standard curve for the column. **C.** Affinity determination through SPR with the RBD of TcsL captured on the chip and a 6-step 5-fold dilution series of each miniprotein starting at 100 nM. Global fit is shown in red while the measured data is shown in black and TcdB binding in gray. **D.** B4 competes with SEMA6A for binding on TcsL. SEMA6A was immobilized on a CM5 chip through amine conjugation. 50nM of TcsL either alone or in the presence of 1000 nM B4 (which should compete) or fzd84 (which should not compete) was flowed over the chip and the binding response plotted.

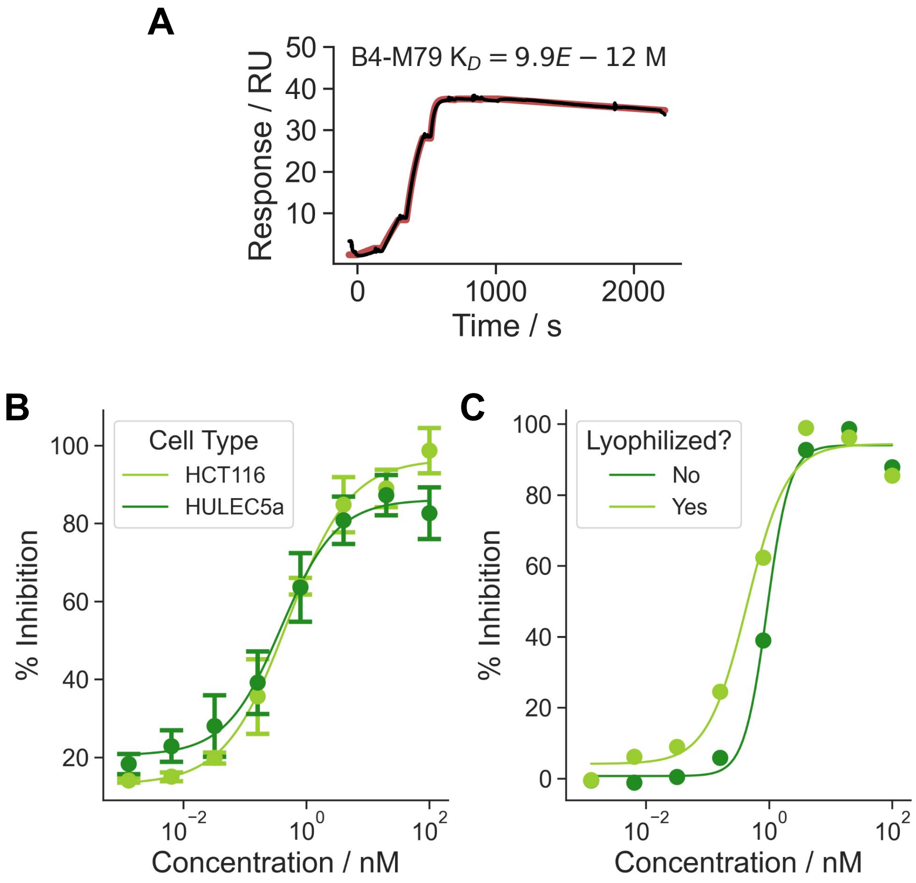

Figure S12. B4-M79 fusions retain binding affinity and neutralization. A. Single cycle kinetic analysis by SPR of B4-M79 fusion with an upper concentration of 200 nM and 6-step 5-fold dilution series. **B.** Neutralization of TcsL in HCT116 and HULEC5a cell lines in the presence of 5 μM human serum albumin with 50 pM and 0.5 pM of toxin, respectively. IC50 of 367 pM (95% CI 177 - 776 pM) for HCT116 400 pM (95% CI 140 - 963 pM) for HULEC5a. Point indicates the mean and error bars are the SEM across 3 independent replicates. **C.** Neutralization of 50 pM TcsL using HCT116 cells with lyophilized and un-lyophilized B4-M79.
